# Are the Traditional Medical Uses of Muricidae Molluscs Substantiated by Their Pharmacological Properties and Bioactive Compounds?

**DOI:** 10.3390/md13085237

**Published:** 2015-08-18

**Authors:** Kirsten Benkendorff, David Rudd, Bijayalakshmi Devi Nongmaithem, Lei Liu, Fiona Young, Vicki Edwards, Cathy Avila, Catherine A. Abbott

**Affiliations:** 1 Marine Ecology Research Centre, School of Environment, Science and Engineering, Southern Cross University, G.P.O. Box 157, Lismore, NSW 2480, Australia; E-Mail: n.nongmaithem.10@student.scu.edu.au; 2 School of Biological Sciences, Flinders University, G.P.O. Box 2100, Adelaide 5001, Australia; E-Mails: david.rudd@flinders.edu.au (D.R.); cathy.abbott@flinders.edu.au (C.A.A.); 3 Southern Cross Plant Science, Southern Cross University, G.P.O. Box 157, Lismore, NSW 2480, Australia; E-Mail: ben.liu@scu.edu.au; 4 Medical Biotechnology, Flinders University, G.P.O. Box 2100, Adelaide 5001, Australia; E-Mails: Fiona.young@scu.edu.au (F.Y.); Vicki.Edwards@bioinnovationsa.com.au (V.E.); 5 Flinders Centre for Innovation in Cancer, Flinders University, G.P.O. Box 2100, Adelaide 5001, Australia; 6 School of Health Science, Southern Cross University, G.P.O. Box 157, Lismore, NSW 2480, Australia; E-Mail: Cathy.avila@scu.edu.au

**Keywords:** ethnomedicine, marine natural products, whelk, indoles, choline esters

## Abstract

Marine molluscs from the family Muricidae hold great potential for development as a source of therapeutically useful compounds. Traditionally known for the production of the ancient dye Tyrian purple, these molluscs also form the basis of some rare traditional medicines that have been used for thousands of years. Whilst these traditional and alternative medicines have not been chemically analysed or tested for efficacy in controlled clinical trials, a significant amount of independent research has documented the biological activity of extracts and compounds from these snails. In particular, Muricidae produce a suite of brominated indoles with anti-inflammatory, anti-cancer and steroidogenic activity, as well as choline esters with muscle-relaxing and pain relieving properties. These compounds could explain some of the traditional uses in wound healing, stomach pain and menstrual problems. However, the principle source of bioactive compounds is from the hypobranchial gland, whilst the shell and operculum are the main source used in most traditional remedies. Thus further research is required to understand this discrepancy and to optimise a quality controlled natural medicine from Muricidae.

## 1. Introduction

Although most natural medicines are derived from plants, marine invertebrate phyla, including the Mollusca, are of increasing interest as a source of novel bioactive compounds [1,2,3,4,5,6]. Marine molluscs are currently used for a range of therapeutic applications, with purified or synthesised bioactive compounds developed as pharmaceuticals and crude or semi-purified extracts as nutraceuticals [4,7,8]. A number of marine molluscs are also used in traditional Chinese, Indian, South African and Middle Eastern medicines [3,9,10,11,12,13,14,15], as well as in homeopathic remedies [16]. Molluscs used directly as a food source may also contribute to the prevention of disease by providing essential nutrients, as well as immuno-stimulatory compounds and other secondary metabolites with direct biological activity [3].

The nutraceutical and functional food industry is currently growing in popularity throughout the world, as an alternative to the pharmaceutical industry [17,18]. However, there is a general lack of scientific data regarding the mechanisms of action of such “complementary and alternative medicines” (CAMs) [19]. Consumers are often under the impression that they must be safe for human consumption simply because they are from a natural source [20]. In most CAMs derived from marine molluscs, the active ingredients are currently unknown and the products have not been tested for efficacy or safety in clinical trials. More information on the toxicology, pharmacology and pharmacokinetics of marine molluscs currently used in CAMs would be highly beneficial. In some cases, independent research on the natural products chemistry and bioactivity of source species may be available. This then provides an opportunity to establish whether the current medicinal uses can be refuted, substantiated, and/or possibly improved on.

This paper reviews the bioactive properties of extracts and secondary metabolites from the Muricidae family of marine gastropods. Muricidae, commonly known as murex or rock whelks, have a long history of pharmacological use, being listed in the Materia Medica by Dioscorides in 1st Century AD, reported by Arabic scholars in 9th Century, and sold in medieval Jewish pharmacies from 11th–14th Century AD [13,21]. A number of Muricidae species are also used in traditional chinese medicine (TCM) [22,23], which has been in use for over 3500 years. The purple secretion from muricids also forms the basis of a homeopathic remedy that has been in clinical use for over 150 years [16,24]. These Muricidae medicines are used to treat a wide variety of disorders, with some re-occurring themes including treatment of menstrual problems, wounds, ulcers and pain relief. However, at least to our knowledge, there are no scientifically rigorous studies testing the efficacy or safety of these CAMs. On the other hand, a substantial body of independent research has been undertaken on the bioactive secondary metabolites and haemocyanins from certain Muricidae species [25,26] and some of these compounds may contribute to the traditional medicinal applications. Here we provide an in depth review of the bioactivity associated with muricid natural products, then outline the current biomedical applications of muricid CAMs and provide an assessment of whether the current CAM applications are potentially substantiated by the presence of pharmacological compounds. This review provides insight into some of the limitations in associating CAMs with bioactive compounds from the source species and highlights the potential for future development of a new scientifically-based nutraceutical from Muricidae molluscs.

## 2. Traditional Muricidae Bioresources

**Figure 1:**
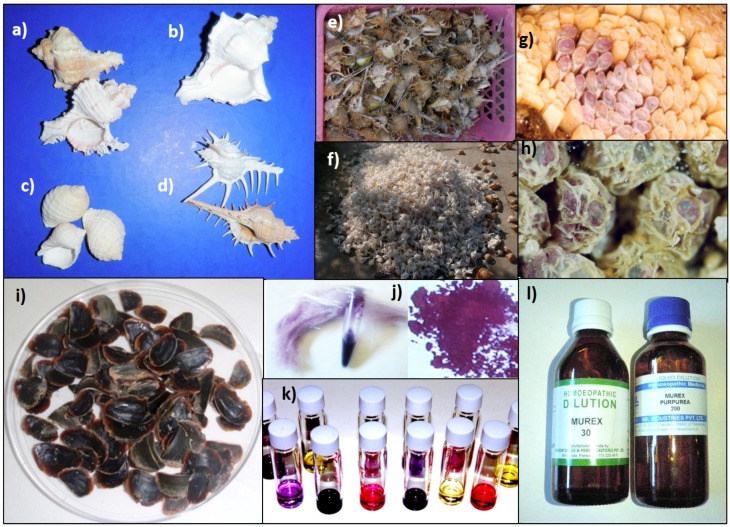
Examples of Muricidae resources: Shell diversity (**a**) *Chicoreus ramosus* (Linnaeus, 1758); (**b**) *Chicoreus virgineus* (Röding, 1798); (**c**) *Dicathais orbita* (Gmelin, 1791); (**d**) *Murex pecten* (Lightfoot, 1786); Harvested Murex (**e**) on sale at a seafood market in Vietnam, and (**f**) processed for the seashell industry in India; Tyrian purple in the egg capsules of (**g**) *D. orbita* and (**h**) *Phycothais reticulata* (Quoy and Gaimard, 1833); (**i**) Operculum from *D. orbita*; (**j**) Tyrian purple; (**k**) indole, indirubin and isatin pigments; (**l**) Murex homeopathic remedy.

**Figure 2:**
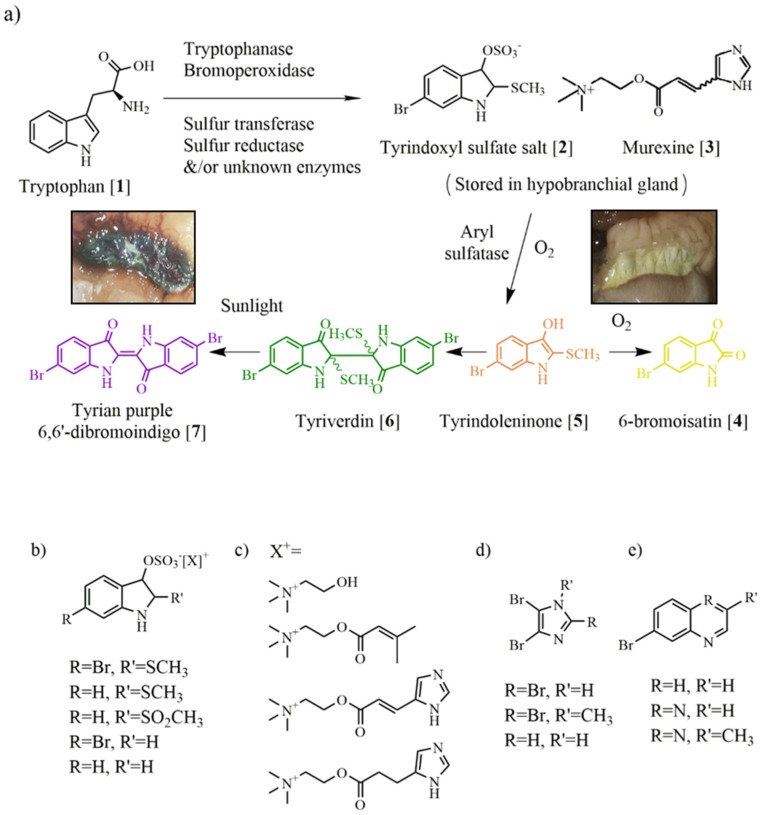
Muricidae chemistry (**a**) showing the proposed formation of Tyrian purple from tryptophan in the hypobranchial glands; (**b**) indoxyl prochromogens; (**c**) choline esters; (**d**) bromoimidazoles and (**e**) bromoquinolines and quinoxoline.

The Muricidae family of marine Mollusca comprises a diverse group of predatory snails, with over 2000 species found across all continents [27]. This cosmopolitan family of marine molluscs is commercially fished for high protein meat throughout Asia, Europe, Central and South America [28,29,30,31]. Many species are highly regarded for their ornate shells (e.g., Figure 1a–d) and operculum (Figure 1i), which is used as an ingredient in incense and some ancient homemade medicines [21,32,33]. They are also highly valued for their purple secretions (Figure 1e,j, Figure 2a), which contain the well-known ancient dye Tyrian Purple [25,34,35,36,37].

A basic awareness of the chemical properties of Muricidae dates back to ancient times [38,39]. The Neolithic Cretans (5000 BC), the Minoans (1600 BC) and ancient Phoenicians and Canaanites, are all thought to have discovered the art of crushing the shells of Muricidae to produce a vibrant purple dye for colouring cloth [38,40,41]. Evidence for a particularly large Muricidae industry is found near Tyre in Lebanon, where there are mounds of broken purple shells [36,40,42]. Numerous civilizations subsequently adopted the ancient industry of purple dye extraction from Muricidae, including the pre-industrial inhabitants of Britain and Norway, people of the West Indies and the Japanese [34,39,41] as well as in Central America, where is it still used by artisans in Mexico and Peru [35,37,43]. More recently, Muricidae have been rediscovered as the original source of *tekhelet*, an elusive sky blue dye mentioned throughout the Hebrew bible Tanakh [44]. The historical importance of these molluscan purple and blue dyes has attracted ongoing interest in the chemistry of Muricidae secretions [25,34,37,45].

Historically the Muricidae whelks were probably fished for food, in addition to their use as a dye source. Hutchinson (1962) found evidence that the Neolithic Cretans and the Minoans not only used the purple dye for colouring textiles, but also consumed species such as *Bolinus (Murex) brandaris* and *Hexaplex trunculus*. The ancient Chinese also exploited Muricidae as a source of purple dye and research has indicated that the Chinese cooked and consumed these snails as part of their diet [40]. The opercula (Figure 1b), used as ingredients in perfume by ancient Mediterranean and Middle Eastern cultures [32,46] may have originally been obtained as a by-product from the fishing and dye industry. The excavation of Bronze Age cargo (dated back to 1300 BC) from the Uluburun shipwreck off the coast of Turkey recovered thousands of Muricidae opercula, thus indicating the development of a significant trade in these unusual muricid resources [47]. From, the eight century onwards, opercula were also exported from Badi near the Red Sea in Sudan [33]. Today whelks from the family Muricidae are still fished all over the world as a food source and/or shell resource, but are less commonly used as a source of purple dye due to the availability of cheap synthetic dyes.

## 3. Muricidae Chemistry

### 3.1. Brominated Indoles and Related Compounds

In the early part of the twentieth century, chemical analysis confirmed the structure of the main pigment of Tyrian purple as 6,6′-dibromoindigo (**7**, Figure 2a) [48]. This uniquely marine metabolite is the brominated derivative of the blue dye indigo, which is also produced by plants in the genus *Isatis* and a range of bacteria [49,50]. Tyrian purple is thought to be synthesised from dietary-derived tryptophan (**1**, Figure 2a) in the hypobranchial glands of Muricidae [25,51]. Baker and Sutherland (1968) isolated the ultimate precursor to Tyrian purple from the hypobranchial glands of the Australian muricid *Dicathais orbita* and identified this as a salt of tyrindoxyl sulfate (**2**, Figure 2a). Four prochromogens including brominated and nonbrominated indoxyl sulfates (Figure 2b) have been identified in other Muricidae [52], and these generate a mixture of purple (6,6 dibromoindigo) and blue pigments, including indigo and monobromoindigo [34]. Baker and Duke (1973) subsequently isolated the intermediate precursors tyrindoxyl and tyrindoleninone (6-bromo-2-methylthio-3*H*-indol-3-one, **5**, Figure 2a), which dimerise to produce tyriverdin (**6**, Figure 2a Christophersen *et al.* 1978), which is photolytically cleaved to produce 6,6′ dibromoindigo (**7**, Figure 2a). A range of nonbrominated indole intermediary precursors have also been identified from the Muricidae extracts [53,54,55,56], as well as oxidative artefacts, including yellow isatins (**4**, Figure 2a) and red indirubins (Figure 1k) [34,45,57].

Whilst the final dye pigments of Tyrian purple are not actually found in the live adult molluscs, chemical studies on the spawn of Muricidae have revealed the presence of Tyrian purple in egg capsules that are hatched or close to hatching (Figure 1g,h) [58,59,60]. The intermediate brominated indole precursors are found in the reproductive organs [61] and egg capsules at earlier stages of embryonic development, suggesting a form of chemical ripening [58]. Over 20 biologically active indole derivatives [62], as well as brominated imidazoles (Figure 2d) [63], brominated quinolines, quinoxalines (Figure 2e) and several unidentified brominated compounds [25], have been identified from the egg masses of various Muricidae species. Studies on the hypobranchial gland and milked glandular extracts of three *Plicopurpura* spp, from Mexico have also revealed seventeen unidentified brominated compounds [37]. Further adding to the chemical diversity in the Muricidae, 6 bromo hydroxyindoles [64] and indolequinones [65] have been isolated from the mid gut of *Drupella fragum*. Indole derivatives are known to have a broad range of pharmacological activities [66,67].

The indole precursors of Tyrian purple can be extracted from the hypobranchial glands, reproductive organs and egg masses using a range of organic solvents, including ethanol, chloroform, dichloromethane, dimethyl formamide (DMF) and dimethylsulfoxide (DMSO) [34,58,61,62]. However, as the intermediate precursors are unstable in oxygen and sunlight, they need to be purified under dark, inert atmospheric conditions (e.g., nitrogen gas). The colourful indole compounds can be separated away from other lipophilic compounds (e.g., fatty acids and sterols) on the basis of their polarity [25] using silica chromatography [58,68,69]. More recently, the intermediate brominated indole precursors have been effectively recovered using CO_2_ supercritical fluid extraction, with partial separation achieved by altering the CO_2_ pressure [70]. The dimeric pigments, such as 6,6′ dibromoindigo, are much more difficult to extract from the molluscan tissue, being insoluble in most solvents, but can be partially recovered by heating in DMSO or DMF [34,61]. The organic extract composition of these Muricidae extracts can be effectively analysed using high performance liquid chromatography (HPLC) or gas chromatography (GC) couple with mass spectrometry (MS) [35,58,61]. Mass spectrometry imaging (MSI) has also been used to investigate the *in situ* distribution of Tyrian purple and precursors in the mollusc tissue [71,72]. These methods will be particularly useful for future biodistribution and pharmacokinetic studies on the bioactive indoles from preclinical *in vivo* animal trials.

### 3.2. Choline Esters

In addition to the indole derivatives, several bioactive choline esters (Figure 2c) have been isolated from polar extracts of the hypobranchial glands of Muricidae molluscs [25,73,74]. In 1976, Baker and Duke discovered the relationship between the choline esters and indoles in the Muricidae by demonstrating that tyrindoxyl sulfate is stored as a choline ester salt [54] and must be hydrolysed by an arylsulfatase enzyme to generate Tyrian purple [57]. These relatively polar choline esters can be easily separated from the lipophylic indoles at the initial extraction phase using polar *vs.* organic solvents or supercritical fluid [70,72].

The most extensively studied choline ester found in the hypobranchial glands of many Muricidae and other neogastropod species, is murexine (**3**, Figure 2a), otherwise known as urocanylcholine or β imidazolyl-4(5)acrylcholine [74]. After the discovery of murexine [75], a number of other choline esters were detected in muricid hypobranchial glands, including dihydromurexine, *N-*methylmurexine, senecioylcholine and the isomer tigloylcholine [74,76,77]. Detection of muricid choline esters mainly involves thin layer chromatography (TLC), with some additional structural elucidation using mass spectrometry and nuclear magnetic resonance [54,72,74,76,77,78]. These choline esters have drug-like properties suitable for oral delivery [25] and have been tested in a number of preclinical and clinical trials for muscle relaxation and toxicity [74].

## 4. Bioactivity of Muricidae Extracts and Compounds

### 4.1. Antimicrobial and Antiviral Activity

Extracts from a number of Muricidae species have been tested for antimicrobial activity against a range of human and marine pathogens, as well as marine bacteria isolated from biofilms (Table 1). Ramasamy and Murugan (2005) undertook a major screening project with a wide range of molluscan extracts against 40 biofilm-forming bacteria isolated from Indian marine substrates. Included in this screening program were whole body extracts from eight Muricidae species, as well as egg mass extracts from four species, digestive gland extracts from two and the operculum from one Muricidae (Table 1). With the exception of digestive gland extracts, all showed inhibitory activity against some of the marine bacterial isolates in at least one of the solvent fractions [79]. The egg mass extracts were the most effective, inhibiting all 40 of the test bacteria (Table 1). The nonpolar solvent extracts used in this study are likely to contain significant amounts of fatty acids and sterol that could contribute to the observed bioactivity. In particular, the whole body extracts from a number of Muricidae appear to be dominated by polyunsaturated fatty acids [80,81], with known antibacterial activity. Conversely, no antibacterial activity was found in a mixture of saturated fatty acids and sterol modelled on the lipid extract composition from the egg mass of several Muricidae species [82]. Benkendorff *et al*., used semi-purified chloroform extracts that concentrated the intermediate Tyrian purple precursors (**4**–**6**) from the egg masses of six Muricidae species (Table 1) in a screening program [83] and examined their ability to inhibit the growth of human pathogenic Gram positive (*Staphylococcus aureus*) and two Gram negative bacteria (*Escherichia coli* and *Pseudomonas aeruginosa*). In all cases, mild inhibitory activity was observed in the chloroform extracts (1–10 mg/mL), whereas no activity was observed in the polar methanol/water fraction (>50 mg/mL) that was dominated by tyrindoxy sulfate (**2**) [83,84]. Several egg mass extracts were also tested against the yeast *Candida albicans*, with similar results [84].

**Table 1:** Antimicrobial and antiviral activity of extracts and compounds isolated from Muricidae. Antibacterial activity tested against human pathogenic bacteria (*Staphylococcus aureus*, *Escherichia coli*, *Pseudomonas aeruginosa*), aquatic pathogenic bacteria (*Vibrio anguillarum*, *Vibrio harveyi*, *Vibrio alginolyticus*, *Enterococcus sericolicida*) or marine biofilm bacteria (panel of 40 isolated strains); Antifungal activity against the yeast *Candida albicans* and antiviral activity against Herpes simplex virus HSV-1.

Source species ^1^	Source Tissue	Extract or Compound	Activity Profile
*Dicathais orbita* [58,83,84]	egg mass	CHCl_3_	Inhibits Gram +ve and Gram -ve human and marine pathogenic bacteria and *C. albicans* in the range of 0.1–1 mg/mL
		Diethyl ether	Inhibits Gram +ve and Gram -ve human pathogens at 10 mg/mL
		EtOH	Inhibits Gram +ve and Gram -ve human pathogens at 0.1 mg/mL
		Tyriverdin	Inhibits human Gram +ve and Gram -ve pathogens at 0.0005 mg/mL, active against *C. albicans* and marine pathogens at 0.001 mg/mL
		Tyrindoleninone	Inhibits human pathogens at 0.5–1 mg/mL, *C. albicans* and marine pathogens at 0.1 mg/mL
		6 Bromoisatin	Inhibits Gram +ve and Gram -ve human pathogenic bacteria in the range of 0.1–1 mg/mL, but >1 mg/mL for *C. albicans* and marine pathogens
*Agnewia tritoniformis* [83,84]	egg mass	CHCl_3_	Inhibits human pathogens at 10 mg/mL
*Bedeva paivae (Trophon hanleyi)* [83,84]	egg mass	CHCl_3_	Inhibits human pathogens at 10 mg/mL
*Ocenebra (Ceratostoma) erinaceus* [63,84]	egg mass	CHCl_3_	Inhibits human pathogens at 1 mg/mL
		2,4,5-Tribromo-1H-imidazole	Inhibits human pathogens at 0.1 mg/mL
*Chicoreus ramosus* [79]	whole body	MeOH	Inhibited 58% of the marine biofilm bacteria tested
	digestive gland	MeOH, H_2_O, DCM, Acetone	No activity against biofilm bacteria
	egg mass	MeOH	Inhibited 100% of the marine biofilm bacteria tested
*Chicoreus virgineus* [79]	whole body	MeOH	Inhibited 50% of the marine biofilm bacteria tested
	digestive gland	H_2_O, DCM or Acetone	No activity against biofilm bacteria
	operculum	MeOH	Inhibited 50% of the marine biofilm bacteria tested
	egg mass	MeOH	Inhibited 50% of the marine biofilm bacteria tested
*Drupella (Cronia) margariticola* [79]	whole body	MeOH/DCM	Inhibited 63% of the marine biofilm bacteria tested
	egg mass	MeOH	Inhibited 70% of the marine biofilm bacteria tested
*Phycothais* *(Lepsiella) reticulata* [83,84]	egg mass	CHCl_3_	Inhibits human pathogens at 10 mg/mL
*Tenguella (Morula) marginalba* [83,84]	egg mass	CHCl_3_	Inhibits human pathogens at 10 mg/mL
*Murex tribulus* [79]	whole body	Acetone	Inhibited 60% of the marine biofilm bacteria tested
*Rapana rapiformis* [79]	whole body	Acetone	Inhibited 23% of the marine biofilm bacteria tested
*Rapana venosa* [85]	haemolymph	Proline rich peptides	Inhibited Gram +ve (*S. aureus)* and Gram -ve (*Klebsiella pneumoniae*)
*Rapana venosa* [86,87]	haemolymph	Haemocyanin	Inhibits the replication of Epstein-Barr virus at 1 μg/mL and Herpes simplex virus type 1 at 200 μg/mL
*Stramonita (Thais) biserialis* [79]	whole body	MeOH	Inhibited 35% of the marine biofilm bacteria tested
*Purpura (Thais) bufo* [79]	whole body	MeOH	Inhibited 25% of the marine biofilm bacteria tested
*Semiricinula (Thais) tissoti* [79]	whole body	MeOH	Inhibited 18% the marine biofilm bacteria tested
*Hexaplex trunculus* [63,84]	egg mass	CHCl_3_	Inhibits *S. aureus* at 1 mg/mL and *E. coli* at 10 mg/mL
		2,4,5-Tribromo-1H-imidazole	Inhibits human pathogens at 0.1 mg/mL

^1^ Accepted species names according to the World Register of Marine Species [88] with previously published genera in brackets.

Bioassay guided fractionation of the egg masses from *D. orbita* has revealed that the brominated indole precursors of Tyrian purple are most likely responsible for the observed antibacterial activity [58]. The fluorescein diacetate (FDA) hydrolysis assay was used to identify the main antibacterial compound as tyriverdin (**6**, Figure 2a), which inhibited the growth of a range of bacteria at 0.5–5 μg/mL. However, the broth dilution assay revealed that this compound is only bacteriostatic and does not lyse the bacterial cells. Mild antibacterial activity (0.1–1 mg/mL) was observed for tyrindoleninone (**5**, Figure 2a) and 6-bromoisatin (**4**, Figure 2a) in the FDA assay, and the broth dilution assay confirmed that these compounds were bacteriolytic [58]. These brominated indoles were also found in egg mass extracts from at least three other Australian and two Mediterranean Muricidae species [62], along with a suite of other non-brominated indoles, which could contribute towards the observed antibacterial activities. The active factors in the Indian species are yet to be identified, however the hypobranchial gland secretions and egg capsules of some of these species, such as *Chicoreus ramosus* undergo colour reactions suggestive of Tyrian purple (KB personal observation), thus implying they also contain the same bioactive indole precursors. Two species of Mediterranean Muricidae, along with *Trophon geversianus* from Chile, were found to contain 2,4,5-tribromoimidazole (Figure 2d) in their egg mass extracts and this compound was also found to inhibit the growth of human pathogenic bacteria at 0.1 mg/mL. The indolequinones from the midgut of *Drupella fragnum* were found to inhibit the growth of *Bacillus subtilis, S. aureus, E. coli* and *P. aeruginosa*, in the range of 7.5 to 50 μg/mL, thus adding to the diversity of antibacterial compounds in Muricidae [65].

The haemolymph of gastropod molluscs contains humoral factors that also provide an important line of defense against microbial pathogens [26,85,89]. The dominant protein in gastropod haemolymph is the oxygen carrying molecule haemocyanin. A glycosylated functional unit of haemocyanin from *Rapana venosa* inhibits the replication of Epstein-Barr virus [86] and *Herpes simplex* virus HSV-1 [87]. *Rapana* haemocyanin has stronger antiviral activity than the haemocyanins from several other marine species, including the well characterised keyhole limpet (KLH1), *Helix vulgaris* and the crustacean *Carcinus aestuarii*. Four proline-rich antimicrobial peptides have also been isolated from *R. venosa* haemolymph [85]. These studies highlight the diversity of mildly antimicrobial agents from different tissues and species in the Muricidae family. Whilst none of the antimicrobial compounds identified so far are active or novel enough to be useful as pharmaceutical drug leads, their broad spectrum of activity could contribute to the medicinal properties of traditional remedies or the consumption of these snails as functional foods.

### 4.2. Wound Healing and Anti-Inflammatory Activity

Muricidae extracts have demonstrated wound healing properties and anti-inflammatory activity in addition to their anti-microbial properties. Lipid extracts from the muricid *R. venosa* were found to significantly improve the healing of induced skin burns in Wistar rats [90]. These lipid extracts contain polyunsaturated fatty acids, Vitamin E, sterols and aromatic compounds; however the specific compounds responsible for conferring wound healing properties have not been identified. Histological analysis revealed that healing time was reduced from 20–22 days in control animals, to just 13–15 days, in mice treated with *R. venosa* lipid extracts [90]. Almost complete regeneration of the skin epidermis, dermis and hypodermis occurred, with new epithelium and newly formed blood vessels, collagen fibres and basal membrane observed in the provisional fibrin matrix. Amino acid extracts from *R. venosa* were also found to accelerate skin wound healing by enhancing dermal and epidermal neoformation in Wistar rats [91]. Healing occurred at least 10 days faster in rats treated with the amino acid extracts compared to untreated controls.

Evidence for the anti-inflammatory activity associated with the lipid extract was supported by normal blood cell counts in experimental rats treated with *R*. *venosa* extracts, compared to increasing quantities of leucocytes, lymphocytes, eosinophils and monocytes in the control rats [90]. The wounds treated with amino acid extracts from *R. venosa* also contained fewer inflammatory cells than the untreated control and the extracts appear to stimulate the proliferation of differentiating keratinocytes [91]. The haemocyanin from *Rapana thomasiana* and *Concholepas concholepas* have been investigated for non-specific immunostimulatory and specific immunomodulatory activity [92,93], with potential adjuvant use in anti-viral vaccination and anticancer therapy. A heparin binding factor with mitogenic growth stimulating activity in T3 fibroblasts has also been isolated from the South American Muricidae *C. concholepas* [94]. Mitogenic heparin binding can promote angiogenesis and increase the rate of dermal repair necessary for wound healing.

Few of the brominated indoles and none of the choline esters isolated from Muricidae have been specifically tested for wound healing or anti-inflammatory activity *in vitro*. However, the bromohydroxyindoles from *Drupella fragum* were found to have anti-oxidant activity in the traditional peroxide value assay [64]. Furthermore, indirubin, a minor pigment in Tyrian purple, blocks the effects of extracellular ATP on macrophages. This prevents the increase in reactive oxygen species (ROS), causing attenuation of phagocytosis and induction of cell death in the presence of ATP [95]. The anti-inflammatory activity of indirubin derivatives have been demonstrated in mouse leukemic monocyte macrophage RAW 264.7 cells [96] and in rat brain microglia [97]. In RAW 264.7 cells stimulated with lipopolysaccharide (LPS), indirubin derivatives inhibited the release of pro-inflammatory cytokines interleukin (IL)-1β and IL-6 [96]. Furthermore, isatin has been found to inhibit inducible nitric oxide synthase (iNOS), cyclooxygenase-2 (COX-2), and tumor necrosis factor (TNF-α), which results in reduced prostaglandin E2 (PGE2) and nitric oxide (NO) levels in mouse macrophages stimulated with LPS and interferon gamma [98]. These isatins, along with some indole derivatives, have been patented for the treatment of inflammation [99,100,101]. Indole derivatives have also been patented for the treatment of osteoporosis [102].

Yazbeck *et al*., [103] recently tested extracts from *D. orbita* (containing tyrindoleninone and 6-bromoisatin) in an *in vivo* rodent model for mucositis (inflammation of the gut). Rats were administered the extract or an oil control by oral gavage for three days, then injected with the chemotherapeutic agent 5-fluorouracil or a saline control and monitored for a further three days. Results from a sugar breath test, histology and myeloperoxidase activity in the small intestine, all indicate that the *Dicathais* extracts alone do not cause inflammation [103]. On the other hand, 5-fluorouracil did cause significant inflammation, but there was no evidence for any gastroprotective effects of the *D. orbita* extracts in rats administered the chemotherapeutic combination.

### 4.3. Anticancer and Kinase Receptor Binding Activity

**Figure 3:**
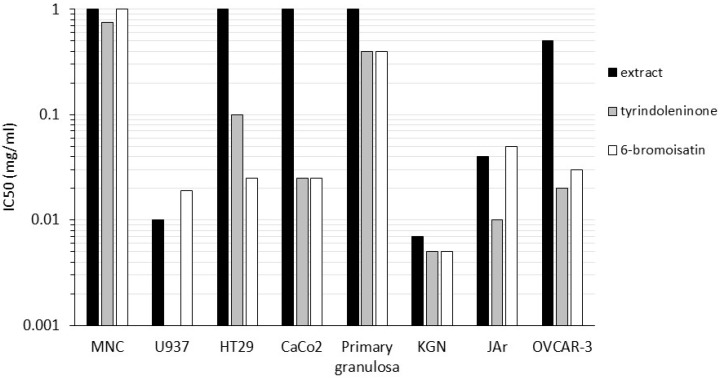
The inhibitory concentration of *Dicathais orbita* crude extract and purified compounds required to reduce cell viability by 50% (IC_50_ mg/mL on a log scale) in a range of cell lines using tetrazolium salt (MTT or MTS) cell proliferation assays; female reproductive cancer cells JAr, OVCAR-3 and KGN, as well as primary derived (healthy) granulosa cells after 4 h exposure [69]; colon cancer HT29 and Caco-2 cells after 12 h exposure [68]; lymphoma U937 and freshly isolated human mononuclear cells after 4 h exposure [51,105]. For freshly isolated human mononuclear cells (MNC), the IC_50_ exceeded the maximum test concentration (1000 µg/mL) for the crude extract and semi-purified fractions after 4 h exposure [104], therefore results are presented for MNC viability after 60 min exposure using Trypan blue staining [126].

The extracts and brominated indoles from Muricidae, as well as a number of synthetic derivatives, show promising anti-cancer activity in a range of *in vitro* and *in vivo* models (Table 2). The chloroform extracts from the egg masses and hypobranchial glands of *D. orbita* inhibit the proliferation of a range of lymphoma and adherent cell lines from solid reproductive and colon tumurs (Figure 3). Bioassay-guided fractionation has identified tyrindoleninone and 6-bromoisatin as the main active constituents inhibiting these cancer cells (Figure 3) [68,69,104,105]. Specificity towards cancer cell lines, relative to the primary derived healthy cells has been observed using these brominated indoles. For example, tyrindoleninone (**5**, Figure 2a) significantly reduced cell viability after just 4 h exposure, in three reproductive cancer cell lines (LC_50_ 0.01 mg/mL KGN and JAr and 0.04 mg/mL OVCAR-3), without significantly affecting primary granulosa cells (LC_50_ 0.9 mg/mL) [69]. Similarly, Benkendorff *et al.* [104] have shown that semi-purified tyrindoleninone inhibited cancer cells (lymphoma cell line U937) at lower concentrations than primary human mononuclear cells (MNC) (Figure 3). This specificity towards cancer cells is a major benefit for any potential application as an anti-cancer drug therapy.

**Table 2:** Anti-cancer and steroidogenic properties of Muricidae extracts. Summary of the (**a**) *in vitro* assays and (**b**) *in vivo* animal models used to investigate the activity of Muricidae extracts and compounds.

**(a) *In Vitro* Assays**		
**Source Species/Compounds**	**Cell Line or Purified Protein**	**Assays ^1^ and Effects Examined**
*Dicathais orbita* (Chloroform extracts, purified tyrindoleninone & 6 Bromoisatin) [51,68,69,104]	A range of female reproductive, colon and breast tumurs and lymphomas (Figure 3)	MTS/MTT cell viability; Crystal violet; Caspase 3/7 activity for apoptosis; Lactate dehydrogenase for necrosis; Tunnel staining for apoptosis; Flow cytometry for apoptosis, necrosis and cell cycle analysis
*Dicathais orbita* (extracts and compounds) [69,106]	JAr and human granulosa cells	Radioimmunoassays (RIA); Steroidogenesis assays: estradiol (E2) and progesterone (P4) synthesis
*Hexaplex trunculus* (purified bromoindirubins) [107,108]	Recombinant or naturally purified protein kinases	CDK1/Cyclin B, CDK5/p25, GSK-3 and other protein receptor kinase assays
*Rapana venosa* (Ethanol extracts) [109]	Human leukemia HL-60 and human lung cancer A-549	MTT cell viability and liquid-scintillation radioassay for cell proliferation (3H-TdR)
*Thais clavigera* (Ethanol extracts) [109]	Human leukemia HL-60 and human lung cancer A-549	MTT cell viability and liquid-scintillation radioassay for cell proliferation (3H-TdR)
*Rapana thomasiana* (Purified haemocyanin) [110]	SiHa-cervical squamous cell carcinoma, CaOV-ovarian adenocarcinoma, MIA PaCa-pancreatic carcinoma, RD 64-rhabdomyosarcoma, EJ-urinary bladder carcinoma and Lep-nontumor human lung cell line.	Cell proliferation assay and apoptosis indicated by DNA degradation and caspase-3 activation
*Rapana venosa* (Haemocyanins) [111]	647-V, T-24 and CAL_29 bladder cancer cells	MTT AND WST-1 cell viability assays, apoptosis with acridine orange/propidium iodine staining and gene expression profiles for 168 inflammatory cytokines and signal transduction pathways.
Synthetic isatin derivatives [105,112]	The human leukemic (U937, monocyte and Jurkat, T cell), breast (MDA-MB-231 and MCF-7), prostate (PC-3), and colorectal (HCT-116)	MTS cell viability, caspase 3/7 for apoptosis, CDK2 inhibition
Synthetic indirubin derivatives [107,108,113,114,115,116]	Recombinant or naturally purified protein kinases	CDK1/Cyclin B, CDK5/p25, GSK-3 and other protein receptor kinase assays; affinity chromatography; crystallography and *in silico* modelling; *rt* PCR on *Xenopus* embryos
Synthetic indirubin derivatives [117,118]	Human neuroblastoma and breast cancer cell lines	Apoptosis induction pathways
Synthetic indirubin derivatives [119,120]	Human melanoma andmyeloid leukemia cell lines	Jak/Stat 3 phosphorylation, FLT3 inhibition
Synthetic isatin and indirubin derivatives [69,106,121]	JAr and human granulosa cells for female hormones and H294 adrenal cells for male	RAI; ELISA for E2 and P4; E-screen (xeno-oestrogenic potential) for E2 receptor binding; H294 adrenal cells for cortisol, testosterone, androgen, and didehydroepiandrosterone
Synthetic indirubin derivatives [122]	JAr and human granulosa cells	RAI
**(b) *In Vivo* Models**		
**Source Species/Compounds**	**Cancer Type**	**Animal Model**
*Dicathais orbita* (Chloroform extracts) [123], (purified tyrindoleninone, 6 bromoisatin) [124]	Colon cancer prevention	Apoptotic response to genotoxic damage by azoxymethane (AOM) in mice. Compounds delivered by oral gavage two weeks prior to AOM
*Concholepas concholepas* (haemocyanin subunits CCHA & CCHB) [92]	Bladder carcinoma treatment	MBT-2 heterotopic murine bladder carcinoma model
Synthetic 6-bromoistain [112]	Colon cancer prevention	Apoptotic response to genotoxic damage by AOM in mice. Compound delivered by oral gavage two weeks prior to AOM
Synthetic 6-bromoindirubin derivatives [119]	Human melanoma treatment	Xenograph model in BALC/c mice, 14 day treatment
Synthetic indirubin derivatives [125]	Renal, prostate, lung and colon cancer treatment	Xenograph model in BALC/c mice

^1^ MTS (3-(4,5-dimethylthiazol-2-yl)-5-(3-carboxymethoxyphenyl)-2-(4-sulfophenyl)-2H-tetrazolium, inner salt), MTT (3-(4,5-dimethylthiazol-2-yl)-2,5-diphenyltetrazolium bromide) and WST-1 (4-[3-(4-iodophenyl)-2-(4-nitrophenyl)-2H-5-tetrazolio]-1,3-benzene disulfonate) are tetrazolium reduction assays for cell viability.

Another promising feature of the Muricidae brominated indoles is that they appear to induce cell death by apoptosis rather than necrosis. KGN, JAr and OVCAR-3 cells incubated with tyrindoleninone were found to undergo morphological changes associated predominately with apoptosis [69]. The up-regulation of caspase-3/7 activity in KGN granulosa cancer cells suggest that both tyrindoleninone and 6-bromoisatin induce cell death by apoptosis at low concentrations [69]. TUNEL staining of the fragmented and condensed nuclei of KGN cells further confirmed activation of apoptosis in the presence of these compounds. Semi-purified 6-bromoisatin (**4**, Figure 2a) also consistently induced caspase-3/7 activity, indicating apoptosis in human colorectal HT29 and Caco-2 cancer cells, while tyrindoleninone tended to induce more necrosis in these cell lines as indicated by LDH release [68]. Flow cytometry using annexin staining confirmed apoptosis in HT29 cells after treatment with ~100 µM of 6-bromoistain, and many of the cells appeared to be arrested in the G2/M phase of the cell cycle [68]. Surprisingly, synthetic 6-bromoistain was not effective in upregulating caspase activity in HT29 colon cancer cells, although the initiation of apoptosis was confirmed by morphological changes in the cells observed under the microscope [112].

Haemocyanin isolated from *Rapana venosa* and *R. thomasiana* have also been found to induce apoptosis in a range of cancer cell lines *in vitro* [110,111]. Gene expression profiling in bladder cancer cells exposed to the haemocyanin revealed significant up-regulation of genes involved in apoptosis, down regulation of some genes involved associated with proliferation and metastatic potential, as well as the activation of inflammatory cytokines [111]. This supports the previously reported immunogenic effects of molluscan haemocyanins, as well as highlighting their potential as chemotherapeutic agents.

The crude extracts and brominated indoles from *D. orbita* have been tested for efficacy *in vivo* in a rodent model for the prevention of colon cancer (Table 2). After 2 weeks of daily administration, the crude extract, at a dose of 1 mg/g, significantly increased the apoptotic response of DNA damaged cells in the distal colon of mice [123]. Using the same rodent model, we reported that synthetic 6-bromoisatin significantly enhanced the acute apoptotic response to the genotoxic carcinogen azoxymethane, at a dose of 0.05 mg/mL [112], suggesting this compound is likely to be the principle anticancer compound in *D. orbita* extracts.

Cytotoxic and apoptotic effects for a range of other natural and synthetic isatin derivatives have been demonstrated against a range of cancer cell lines [67,105,127,128]. Of particular note, the minor pigment of Tyrian purple 6, 6-dibromoindirubin was found to be a potent inhibitor of glycogen synthase kinase-3 (GSK-3) [113]. Indirubin has been chemically synthesized and modified to produce a range of synthetic derivatives with anti-proliferative properties [107,108,129]. These indirubin derivatives induce apoptosis in lung, stomach, colon, abdominal and leukaemia cancer cell lines (Table 2). The synthetic derivatives also have improved solubility and biological selectivity [107,115,130,131], inspiring interest as pharmaceutical agents for the treatment of cancer and leading to several patents over the last decade [132,133,134,135,136]. *In vivo* studies on the isatin derivative AGM011 indicate that this compound can suppress solid tumurs in rats by 80% [133]. A number of indirubin derivatives are currently used for treating leukaemia, lung and prostate cancer [114,130,131].

### 4.4. Steroidogenic Effects of Muricidae Extracts and Analogue Compounds

The ongoing use of Muricidae traditional medicines to treat Women’s problems (detailed in Section 5 below) implies possible effects on steroidogenesis. Steroid hormone production is critical for maintaining the menstrual cycle, for development and production of mature oocytes, and for establishing and maintaining pregnancy [137]. In women, the steroidogenic pathway is expressed within the granulosa cells of the ovary, and in uterine and placental cells. The human JAr chorioplacental cancer cell line and primary-derived granulosa cells from healthy women, express functional steroidogenic pathways *in vitro*. They respond to human chorionic gonadotrophin (hCG) *in vitro* by upregulating steroidogenesis and increasing the levels of progesterone and estradiol secreted into the cell culture medium [106,138].

Edwards *et al*., tested the effects of *D. orbita* extracts, containing tyrindoleninone and 6-bromoisatin, on cytotoxicity, progesterone (P_4_) synthesis and hCG responsiveness in the JAr cell line [106]. The *D. orbita* extract significantly decreased JAr cell viability, with the IC_50_ affected by incubation time, but not hCG. Using a radio-immunoassay, this brominated indole-containing extract was found to inhibit hCG-stimulated P_4_ production at low concentrations (0.005 mg/mL), but stimulated P_4_ at the highest concentrations (0.5 mg/mL), after taking into consideration the cytotoxic effects [106]. Further testing using two synthetic indirubins and 5-bromoisatin revealed similar responses in the JAr cancer cell line; with a hormetic n-shaped response, in which low doses stimulated but high doses had no effect on constitutive progesterone synthesis [138]. Hormesis or a low-dose enhancement, with a dual or biphasic effect, has been previously observed for the effects of natural products on steroidogenesis. For example, imidazoles induce a biphasic response on adrenal hormone synthesis [139]. Typically steroidogenesis is stimulated at low doses and inhibited at high doses. In contrast, *D. orbita* extracts appear to cause an unusual inverse hormetic response on P_4_ synthesis (*i.e*., inhibition at low doses and stimulation at high doses over a narrow dose range) [106].

Edwards *et al*., have also tested the effects of *D. orbita* extracts and compounds on the production of progesterone and estradiol in granulosa cells, with and without hCG [138] (Table 3). In addition, several synthetic isatin and indirubin compounds were tested for effects on basal progesterone production in granulosa and JAr cells (Table 3). Cytotoxicity affects steroidogenesis and secretion, therefore only non-cytotoxic doses of Muricidae compounds are considered in Table 3. The comparison of semi-purified compounds with crude *D. orbita* extracts suggests a specific and targeted activity for certain brominated indole derivatives (Table 3). It appears that tyrindoleninone either has no effect, or is inhibitory towards basal and hCG-stimulated progesterone synthesis, but elicits an n-shaped dose response curve with respect to basal and hCG-stimulated estrogen synthesis. Low, but not high concentrations of tyrindoleninone stimulated estrogen synthesis, but did not affect progesterone synthesis. This suggests that tyrindoleninone may act on targets in the estrogen biosynthetic pathway subsequent to progesterone production. The granulosa cells responded to indirubin and 5-bromoisatin differently from the JAr cells [138], although the dose-dependent stimulation caused by 5-bromoisatin may have been equivalent to the stimulatory phase of the JAr cell n-shaped dose response. The effects of 6-bromoisatin on granulosa cell steroidogenesis were variable, and more data are required to characterise the steroidogenic effects of this compound (Table 3). Nevertheless, it is clear from these preliminary studies that Muricidae extracts and compounds do have the potential to moderate or interfere with steroidogenesis.

**Table 3:** Effect of Muricidae extracts and synthetic analogue compounds on basal and gonadotrophin-stimulated progesterone and estrogen synthesis *in vitro*. Only the effects of non-cytotoxic concentrations of compounds are summarised in this table.

Source	Compound	Cell Type	Hormone	Dose Response
Synthetic compounds 0, 0.00001, 0.0001, 0.001, 0.01, 0.1 mg/mL	6,6′ dibromoindirubin	JAr	Basal P4	N-shaped ^1^, low doses stimulated, 0 and high doses N/E, 4 h, 6 h, 8 h, 10 h
	indirubin		Basal P4	N-shaped, Low doses stimulated, 0 and high doses N/E, 24 h
	5-bromoisatin		Basal P4	N-shaped, low doses stimulated, 0 and high doses N/E, 24 h
	indirubin	GC	Basal P4	U-shaped ^2^, 48 h, 72 h
	5-bromoisatin		Basal P4	Dose-dependent stimulation, 48 h
Hypobranchial gland extract 0, 0.005, 0.01, 0.05, 0.1, 0.5, 1 mg/mL	mixture of 6-bromoisatin, tyrindolinone, tyrindoleninone, tyrindoxyl sulphate	GC	Basal P4	N/E
			hCG P4	N/E
			Basal E2	0.05 mg/mL stimulated, 24 h
			hCG E2	U-shaped, 4 h, 24 h, 48 h
	tyrindoleninone	GC	Basal P4	N/E
			hCG P4	N/E
			Basal E2	N-shaped, 24, 48 h
			hCG E2	N-shaped, 24, inhibition—48 h
	6-bromoisatin	GC	Basal P4	U-shaped 4 h, N/E 24 h, 48 h
			hCG P4	N/E
			Basal E2	U-shaped 4 h, N/E 24 h, 48 h
			hCG E2	Dose-dependent inhibition 4 h, 48 h
Egg mass extract 0, 0.005, 0.05, 0.5 mg/mL	mixture of 6-bromoisatin, tyrindolinone, tyrindoleninone, tyriverdin	GC	Basal P4	N/E
			hCG P4	Dose-dependent stimulation, 48 h
			Basal E2	N/E
			hCG E2	Dose-dependent stimulation, 48 h
	tyrindoleninone	GC	Basal P4	N/E
			hCG P4	Dose-dependent inhibition
			Basal E2	N-shaped 24, 48 h
			hCG E2	Stimulation 4, 24 h, n-shaped 48 h
Egg mass extract 0, 0.005, 0.05, 0.5 mg/mL	6-bromoisatin	GC	Basal P4	Lowest dose cytotoxic
			hCG P4	Dose-dependent inhibition, 48 h
			Basal E2	Lowest dose cytotoxic
			hCG E2	Dose-dependent stimulation, 48 h

JAr—human chorioplacental cancer cell line, GC—human primary-derived granulosa cells, P4—progesterone, E2—estradiol 17 beta, basal—constitutive hormone synthesis, hCG—exposed to human chorionic gonadotrophin *in vitro*, N/E—no effect and the same as control, stimulation—steroid hormone synthesis stimulated to be significantly higher than controls, inhibition—steroid hormone synthesis significantly lower than controls, h—hours of exposure *in vitro* [106,136].^1^ N shaped curve = typical hormetic response with low dose stimulation and high dose inhibition; ^2^ U shaped response = inverse hormesis with low dose inhibition and high dose stimulation.

### 4.5. Muscle Relaxing and Nicotinic Activity of Choline Esters

Hypobranchial gland extracts from a number of muricid species have shown potent muscle relaxing properties with nicotinic action, of which choline esters are the major contributors [74,140,141] (Table 4a). The choline esters are found in the polar fraction of organic solvent extracts and are soluble in ethanol, methanol, acetone and to some extent water [72,74]. Some muricid species are known to contain multiple choline esters (Figure 2c), predominantly from the hypobranchial gland [74]. Similarities in the pharmacological properties of the gland extracts and choline esters have been demonstrated *in vitro* using the frog rectus assay (Table 4a) [74]. These compounds elicit neuromuscular blocking action, but do not bind to muscarinic acetylcholine receptors, correlating with affinity to nicotinic cholinergic ligand-gated ion channels [141]. Keyl and Whittaker (1958) were able to measure depolarization on the endplate region of rat gracilis muscle at concentrations of 3 mg/kg, whereby murexine may be binding to nicotinic acetylcholine receptors. The concentration dependent effect of murexine on twitch reduction has also been tested *in vitro* on cat, dog and rabbit gastro-cnemius [140] (Table 4a)*. In vivo* studies using murexine, dihydromurexine and senecioylcholine support the neuromuscular blocking activity seen *in vitro* in the frog rectus assays (Table 4a). All three choline esters produced head drop in rabbits and paralysis of the back legs in dogs [140]. Clinical trials on 160 human patients after intravenous (i.v.) administration of murexine demonstrated paralysis lasting for 3–6 min after a single dose of 1 mg/kg, whereas longer lasting muscular relaxation was achieved using slow i.v. infusion of a 1/1000 solution of murexine in physiological saline [140].

**Table 4:** Neuromuscular and pain signalling effects of Muricidae extracts and compounds; (a) Choline ester and hypobranchial gland (HG) muscle relaxing and nicotinic activity; (b) Isatin derivative neurotransmitters, analgesics and sedative properties of the Muricidae extracts and compounds.

**(a)**						
**Source Compound ^a^**	**Conc.**	**Assay/*in Vivo* Model**				**Observed Effects**
**HG Extract/Synthetic**						**Neuromuscular block** Paralysis of the skeletal musculature after intravenous (i.v.) administration. Murexine stimulates nicotinic acetylcholine receptors opening the monovalent cation channel for depolarization of the motor endplate. Mild or no muscarine like activity was detected in guinea-pig and rabbit intestine, rabbit atrium and isolated frog heart assays.
M, MCH, DHM, SCH	various	Frog rectus abdominis muscle assay [74,76,140]				
M, MCH	0.75 mg/assay	Neuromuscular block in rat diaphragm assay [140]				
M, MCH	100–2000 μg/kg	% Twitch reduction assays on cat, dog and rabbit gastro-cnemius were used to calculate concentration effect [75]				
M, MCH	0.65 mg/kg	50% rabbit head drop after i.v. injection [74]				
DHM	0.52 mg/kg					
M, MCH	0.35 mg/kg	50% dog paralyzing dose after i.v. injection [140]				
DHM	0.022 mg/kg					
M, MCH	1.0–1.2 mg/kg	Relaxing effect in human preliminary clinical trial on 160 patients after a single i.v. injection [140]				
**HG Extract/Synthetic**						**Nicotinic activity** Murexine i.v. at high dose is likely to have nicotinic effects on sympathetic ganglia and adrenal medulla
M	60 μg/kg/min (no effect)	Nicotinic effects of murexine showed a dose dependant rise in blood pressure whilst inducing neuromuscular block in anaesthetised cats and dogs [74,140,141]				
M	300 μ/kg					
**(b)**						
**Compound**	**Conc.**	**Assay/*in Vivo* Model and Effects Observed**	**Method Admin.**	**Mode of Action/Pathways Identified**	**Overall Effects**	
2,3 dioxoindoline	15–20 mg/kg	Mice and rats showed anxiogenic behaviour in the open-field and elevated plus maze test and the social interaction test [142]	i.p.	Monoamine oxidase B inhibition as a contribution to stress related tribulin activity.	Anxiogenic at low dose	
	<50 mg/kg	Mice showed immobility in the forced swim test [143]	i.p.	Inhibits monoamine oxidase affecting monoamine levels.	Sedative at high dose	
indole-2,3-dione	20 mg/kg	Isatin administered after pentylenetetrazole (PTZ) and 3-mercaptopropionic acid (3MPA) induced seizures in rats [143]	i.p.	Antagonise natriuretic peptide receptor A (NPR-A) and NPR-C signalling at low dose due to the metabolite 5-hydroxyisatin.	Proconvulsant at low dose	
	60–80 mg/kg	Effective against PTZ and 3MPA induced clonic convulsions [144]	i.p.		Anticonvulsant at high dose	
indole-2,3-dione-3-oxime or as salt, oxide or hydrate derivative	10 uM	Intermediate-conductance and small-conductance Ca(2+)-activated potassium channel (IKCa and SKCa)activation in a 15 ul cell chamber on human embryonic kidney 293 cell line [145]	immersion	Ikca and SKca ion channel associated conditions including respiratory conditions, muscle spasms, convulsive conditions, mood disorders and dementia.	Ikca and Skca ion channel activation	
5,7-dinitro-1-methyl-1H-indole-2,3-dione-3-(0-methyloxime)	0.1–10 mg/kg	Administered to NMRI mice for ATPA rigidity, to DAB/2 mice for quisqualate seizures, to NMRI mice for *N*-methyl-d-aspartate (NMDA) seizures and to NMRI mice for cocaine hypermotility [146]	i.v. and orally (cocaine hypermotility)	Excitatory amino acid antagonist blocking glycine and glutamate on the quisqualate, 2-amino-3-(3-hydroxy-5-tert-butylisoxazol-4-yl)propionic acid (ATPA), 2-amino-3-hydroxy-5-methyl-4-isoxazole propionic acid (AMPA), kainate and NMDA receptors.	Anticonvulsant for ATPA quisqualate, NMDA seizures and cocaine hypermotility	
5-bromoisatin	200 mg/kg	Phenylquinone test for analgesia in mice showing 90% inhibition after 30 min [147]	i.p.	5-bromoisatin was comparable to acetylsalicylic and showed analgesia with fewer side effects.		Analgesic
	400 mg/kg	Randall and Selitto test for analgesic comparison in rats [147]	orally			
	90 mg/kg	Overall ED50 after 30 min reaction time [147]				

^a^ M—murexine, was extracted from *Murex trunculus*, *Murex brandaris* and *Tritonalia erinacea*; MCH—murexine chloride hydrochloride (synthetic); DHM—dihydromurexine; and SCH—senecioylcholine are from hypobranchial gland extracts of the above species. Administration method includes: i.p.—intraperitoneal and i.v.—intravenous.

When compared to other muscle relaxants, including decamethonium, suxamethonium, gallamine and tubocurarine, the muricid choline esters show greatest similarity in dose and effect to suxamethonium, another depolarizing type of neuromuscular blocker [140]. There are a number of features of the structure of murexine (**3**, Figure 2a) and other muricid choline esters that influence the potency of their activity as muscle relaxants, including the electron density of the “ether” oxygen atom and the quaternary ammonium ion (O(CH_2_)_3_N^+^(CH_3_)_3_ group) (Figure 2c), which has similarity to acetylcholine [148]. These structural features may explain the nicotinic activity demonstrated by the muricid choline esters, as they appear to mimic the action of acetylcholine on nicotinic acetylcholine receptors, although this interaction is yet to be confirmed. *In vivo* nicotinic activity is dose dependent; with low doses having little effect on respiration and blood pressure [74], whilst high doses above 100–200 mg/kg increase blood pressure and respiration in anaesthetized cats and dogs [141], indicating sympathetic ganglion stimulation in addition to neuromuscular blocking activity. The consequences of the nicotinic activity in humans are side-effects such as nausea and vomiting. This was observed in some subjects in the clinical trial using murexine, thus deterring further clinical studies or applications with this compound [140].

### 4.6. Neurotransmitters, Analgesic and Sedative Properties of Isatins

Isatin is an endogenous oxidized indole which has a wide range of effects in mammalian systems, including behavioural and metabolic functions [149]. As such, synthetic and naturally derived isatin derivatives also exhibit a diverse range of effects, including anxiogenic, anticonvulsant, sedative, analgesic activity and ion channel activation (summarized in Table 4b). Endogenous isatins which are known to increase during stress have their greatest potency as antagonists of atrial natriuretic peptide (ANP) function and nitric oxide (NO) signalling, both of which are potent vasodilators [149]. When isatin is administered in rat models of Parkinson disease it inhibits monoamine oxidase B and improves bradykinesia and striatal dopamine levels [128]. *In situ* imaging of [^3^H]isatin in rat brains has demonstrated its distribution in the hypothalamic nuclei, cortex, hippocampus and cerebellum [150]. Therefore, any exogenous isatin and derivatives based therapeutic agents should be carefully administered as they may influence the many endogenous isatin targets, if they are capable of crossing the blood brain barrier [151].

Synthetic 5-bromoisatin, has demonstrated analgesic effects at high doses [147], similar to synthetic isatin [144]. This compound is a structural isomer of 6-bromoisatin found in Muricidae extracts. Further investigations of 6-bromoisatin in comparison to synthetic isatins are required to establish any interaction with endogenous isatin targets. For example, 6-bromoisatin may show potential as a neuromodulator with effects on monoamine oxidase B, ANP function and NO signalling.

### 4.7. *In Vivo* Toxicity

As choline esters bind to nicotinic acetylcholine receptors, resulting in neuromuscular blocking activity, extracts from the hypobranchial glands of muricid molluscs could have a certain level of toxicity associated with their administration. Table 5 compares the *in vivo* toxicity of the various muricid choline esters. The lethal effect of muricid choline esters appears to be attributed to the combined neuromuscular and nicotinic activity. Tigloylcholine administered i.v. to mice (0.92 mg/kg) produced tonic tremors, convulsion and jumping, symptomatic of nicotinic receptor stimulation [77]. This resulted in death after two minutes due to respiratory arrest, possibly from neuromuscular blocking of muscles associated with respiration [77]. Tigloylcholine shows a higher potency compared to murexine and dihydromurexine, but as tigloylcholine appears to be an isomer of seneciolylcholine [77], differences between these two choline esters are difficult to compare without further confirmation of the specific structures used in *in vivo* experiments. Further chemical studies using nuclear magnetic resonance are required to distinguish tigloylcholine from seneciolylcholine and other choline esters for use in future pharmacological studies [25,77].

Recent *in vivo* studies have also been undertaken to assess the gastrointestinal and hepatotoxicity of brominated indoles and lipophylic extracts from *D. orbita* (Table 5). In a two week toxicity study in mice, there was no mortality, clinical toxicity symptoms or weight loss resulting from the administration of the semi-purified extract [152]. Similarly no mortality or clinical toxicity was observed during two or 15 week colon cancer prevention models in mice [122,123,153]. However, histological examination after a two week toxicity trial revealed idiosyncratic effects on the gastrointestinal tract and liver, in a small proportion (<40%) of mice administered the extract, including necrosis, fatty change, and inflammation [152]. In the two week colon cancer prevention model, liver enzyme levels in the mice were not affected by the crude extract, semi-purified 6-bromoisatin [124] or synthetic 6-bromoisatin [112]. However, aspartate aminotransferase serum levels in the mice treated with tyrindoleninone were elevated significantly compared to the saline control [124] indicating potential hepatotoxicity. Tyrindoleninone also appeared to reduce the red blood cell counts and haemoglobin levels in the mice suggesting mild anaemia. However, tyrindoleninone is likely to be converted to 6-bromoistain in the stomach, as indicated by a treatment of the crude extract with simulated digestive fluid [123]. 6-Bromoisatin does not appear to have any significant side-effect on the blood cells or serum biochemistry, with the exception of a reduction of potassium levels indicating possible diuretic effects [112,124]. This is consistent with previous reports of diuretic effects associated with isatin derivatives by Nataraj *et al.* [154]. Overall, the crude muricid extract dominated by 6-bromoisatin and synthetic 6-bromoisatin appear to be relatively safe for oral ingestion. Nevertheless, purification and strict quality control would be required to ensure the effective separation of the more toxic choline esters from any natural medicines derived from the hypobranchial glands of Muricidae.

**Table 5:** Toxicity of Muricidae compounds and extracts, indicating general effects (GE) and lethal dose (LD).

Compound/Extract	Model	Test	Method Admin	Conc. (mg/kg)	General Side Effects
murexine	Mouse	LD_50_	i.v.	6.45	Paralysis of the skeletal musculature preceded by transient stimulation including muscle tension and fasciculation. Death is caused by anoxia after peripheral respiratory arrest [74]
		LD_50_	s.c.	50	
		LD (ineffective)	oral	<1000 ineffective	
	Dog	GE	i.v.	0.27	For dogs, murexine additionally caused increased saliva and evacuation of urine and faeces (defecation). Nevertheless they handled 200 times dose with artificial respiration (intubation) [74]
		GE	s.c.	1.35–2.16	
	Pigeon	GE	i.v.	0.05	Birds developed contracture, leg cramp and opisthotonus instead of muscular paralysis [74]
		LD_50_	i.v.	0.2–0.3	
	Octopus	GE	i.b.h.	30–40	*Eledone moschata* showed brief stimulation and motor agitation with deep respiratory behaviour [74]
	Humans	GE	i.v.	1–1.2	Muscle relaxation with mild nicotinic effects [140]
dihydromurexine	Mouse	LD_50_	i.v.	5.57	Similar to murexine but more potent for mice. 12 times more potent for frog rectus abdominis [74]
tigloylcholine	Mouse	LD_50_	i.v.	0.92	Considerably more potent than murexine [77]
*D. orbita* brominated indole extracts	Mouse	GE	oral	0.5	Idiosyncratic effects on hepatocytes including nonsteroidal fatty change and necrosis [152]
		GE	oral	0.5	Idosyncratic gastrointestinal inflammation and ulcers [152]
		LD (ineffective)	oral	>1	No mortality after 4 weeks daily oral gavage, no effects on behaviour or any signs of ill health [153]
	Rat	GE	oral	1	No mucositic, inflammation, or negative effects of gastric epithelium or blood cells [103]
		LD (ineffective)	oral	>1	No mortality after 1 week daily oral gavage, no effects on behaviour or any signs of ill health [103]
6-bromoisatin	Mouse	GE	oral	0.25–1	Diuretic effects evidenced by reduced K/Na ratio in blood no negative effects on blood cells, hemoglobin or serum liver enzymes [112]
		LD (ineffective)	oral	>1	No mortality after 2 weeks daily oral gavage, no effects on behaviour or any signs of ill health [112]

LD_50_—lethal dose for 50% of group; LD (ineffective)—tested for safety with no mortality; i.v.—intravenous injection; s.c.—subcutaneous injection; i.b.h—intra branchial heart.

## 5. Traditional Medical Uses

### 5.1. Ancient Mediterranean and Middle Eastern Use

Early records of natural medicine state that the operculum, flesh and shell of neogastropods were all used for their therapeutic properties [21]. The operculum from Muricidae were used for curing a range of illnesses, such as swollen spleen, depression, rheumatism or arthritis, stomach ulcers, skin diseases including boils, warts and tumors, teeth problems, eye disease, hearing loss, epilepsy and paralysis (Table 6). These opercula are also reported to be useful as purgatives and laxatives [13,14,15,155]. The opercula were also specifically used for the treatment of female reproductive disorders including menstrual cycle abnormalities, atresia of the uterine cervix and other diseases of the uterus, as well as for removal of the placenta after labour (Table 6). The opercula are still reported to be important ingredients in traditional Sudanese perfumery such as *karkar*, *dukhan*, *dilka*, *khumra* and *bakhur mu’assal* [33]. The uses of these perfumes containing opercula have strong connection with marriage and only brides or married women can apply them. *Khumra* are reported to help make sexual intercourse easier, especially for the first night, whereas *dukhan* tightens the vaginal muscles and is suggested to cure women’s diseases. To date, there appear to be no chemical studies on the opercula of neogastropods to help explain these applications. However, the analgesic and anti-inflammatory isatins (Table 4b) and muscle-relaxing choline esters found in Muricidae (Table 4a), along with the steroidogenic properties of the extracts and indole derivatives (Table 3) could feasibility contribute to some of these applications, providing these bioactive compounds are both present and biologically available in these traditional medicinal preparations.

The burnt flesh and ashes of muricid shell were reported to have anti-inflammatory properties and were traditionally used for wound healing, cleaning teeth, treatment of cracked skin and healing parotid gland swelling [21] (Table 6). The boiled foot muscle was used specifically to heal stomach ulcers. These applications are consistent with anti-inflammatory properties of isatin derivatives, as well as the anti-bacterial (Table 1) and wound-healing properties of Muricidae extracts. The purple secreting hypobranchial glands of Muricidae were also used in medieval times, as laxatives and diuretics [21] (Table 6), which is consistent with the presence of choline esters and 6 bromoisatin, respectively. This gland was also said to increase perspiration and salivation and cause nausea and vomiting if used in excess (Table 6). These side effects are consistent with the nicotinic receptor binding activity of choline esters, as observed in clinical trials using murexine [74].

**Table 6:** Ancient medicinal uses of Muricidae molluscs.

Culture	Part of snail	Source species	Preparation	Pharmaceutical properties (Treatment)
Ancient Greco-Roman (Dioscorides, Oribasius and Galen) [21]	Operculum	*Hexaplex trunculus*, *Bolinus brandaris*, *Thais haemastoma*	Crushed and mixed with oil and vinegar	Hearing loss, swollen spleen, depression, menstrual cycle abnormalities, after labour for placenta removal
	Flesh and ashes of burned shell	*Hexaplex trunculus*, *Bolinus brandaris* and *Thais haemastoma*	Burned flesh along with shell	Wound healing, cleaning teeth, treatment of cracked skin, healing parotid gland swelling, anti-inflammatory properties
Ancient Greece (Dioscorides) [155]	Whole shell with meat	Muricidae (*Purpura-*Tyrian Purple producing shellfish)	Burnt and dry whole animal	Cleaning teeth, healing warts, boils or tumour
	Columellae	“Purpurae” (Muricidae)	Burnt and dry	Good for stomach
Ancient Greece (Xenocrates) [21]	Hypobranchial gland	*Hexaplex trunculus*, *Bolinus brandaris* and *Thais haemastoma*	unknown	Laxative, diuretic, increases salivary secretion, perspiration.Excess consumption may cause nausea, vomiting and diarrhoea
Ancient Greece (Athenaeus) [21]	Foot	*Hexaplex trunculus*, *Bolinus brandaris* and *Thais haemastoma*	Boiling	Heals stomach disorders
Medieval Eastern Mediterranean Genizah [14,155]	Operculum	Muricidae such as *Murex anguliferus*	Smell the aromatic substance or smoke produced while placing the operculum on slowly burning charcoal	Rheumatism or arthritis Stomach problem (wounds in stomach), skin diseases, teeth problems, eye and ear diseases, tumors, epilepsy , paralysis, purgative, treatment of diseases of the uterus
Bahrein Middle Eastern [13,15]	Operculum	*Murex inflatus*	Fumigation	Atresia of uterus
Europe (Aphrodisiacs) [156]	Operculum *(Blatta byzantine)*	Banded dye Murex	Operculum medicine with vinegar Fumigation	Reduced swollen spleen For women (dislodge the placenta after labour)
	Operculum	Calcified operculum	Ashes of calcified operculum	Stimulate capillary growth

### 5.2. Muricidae Used in Traditional Chinese Medicine (TCM)

Many species from the Muricidae family have been used in TCM to treat various diseases (Table 7), although little rational scientific evidence for their efficacy can be identified. Some traditional uses such as for *Rapana bezoar* and *R. venosa*, and *T. clavigera*, have been summarised and clarified in “Ben Cao Gang Mu” or “Compendium of Materia Medica” (1578 A.D.). However, the traditional uses of other Muricidae species are spread over a number of books. Fortunately, all of the information has been systemically summarised to two recent Chinese books, “Zhong Hua Ben Cao” or “The Chinese herbal” [23] and “Zhong Hua Hai Yang Ben Cao” or “Chinese Marine Materia Medica” (Guan and Wang, 2009). The traditional uses of Muricidae species introduced here are mainly based on the information retrieved and translated from these two recent books.

**Table 7:** Uses of different Muricidae family species in Traditional Chinese Medicine (TCM) ^1^.

Scientific Name	Method of Use	Traditional Uses and Claims
**Genus *Ceratostoma***		
*C. rorifluum* (Adams & Reeve)	Decoct the shell (10–50 g) and ingest.	Tranquilize and sedate the mind; astringe and preserve the essence; Use to treat insomnia, amnesia, spermatorrhea, uterine bleeding and leukorrhagia.
**Genus *Chicoreus***		
*C. asianus* (Kuroda)	Same as *C. rorifluum*	Same as *C. rorifluum*
*C. brunneus* (Link)	Decoct the shell (15–25 g) and ingest.	Resolve phlegm, disperse retention, tranquilize liver and wind; Use to treat stomach pain, scrofula and spastic muscles.
*C. ramosus* (Linnaeus)	Decoct the crushed shell (15–50 g); Ustulate (scorch) the shell, ground into powder and apply externally.	Clear heat, expel toxins, soften hard lumps, dispel nodes, reduce flatulence and pain; Use to treat pathopyretic ulcer, scrofula (infection of the lymph nodes), stomach pain, dyspepsia, stomach and duodenal ulcer.
**Genus *Murex***		
*M. aduncospinosus* (Beck) ^2^	Decoct the shell (15–25 g) and ingest. Ustulate the shell, ground into powder and apply externally.	Clear heat, expel toxins, invigorate blood circulation. Use to treat pyretic toxicity, carbuncle, furuncle, otitis medium and ulcer of lower limb.
*M. pecten* (Lightfoot) ^3^		
*M. rectirostris* (Sowerby) ^4^		
*M. ternispina* (Lamarck)		
*M. trapa* Röding		
**Genus *Nassa***		
*N. francolinus* (Bruguière) ^5^	Same as genus *Murex*	Same as genus *Murex*
**Genus *Purpura***		
*P. rudolphi* (Lamarck)	Same as *T.* alouina, *etc.*	Same as *T. alouina*, *etc.*
**Genus *Rapana***		
*R. bezoar* (Linnaeus)	Fresh meat: boil and eat the meat; decoct the shell (30–60 g) and ingest. Combine the juice and with other medicine as eye drops	Fresh meat: Remove heat to brighten vision; Use to treat hepatic heat and red eyes, ophthalmalgia, chest and abdomen heat and pain.
*R. bezoar* (Linnaeus)	Shell: Decoct the shell (15–30 g) and ingest, used as medicinal powder (3–6 g) and ingest; Ustulate the shell, ground into powder, mixed with sesame oil and apply externally. Operculum: Decoct the operculum (10–20 g) and ingest; Ustulate the shell, ground into powder and apply externally.	Shell: Relieve gastric hyperacidity to alleviate stomachache, resolve phlegm, disperse retention, tranquilize liver and wind; Use to treat stomach and duodenal ulcer, panasthenia, spastic hand and foot, chronic osteomyelitis, and scrofula. Operculum: Clear heat, expel toxins, remove dampness through diuresis, free strangury. Use to treat strangury (painful & frequent urination), swelling and ulcer on the body surface, hepatic coma, eye diseases, dysentery.
*R. rapiformis* (Born)	Decoct the shell (15–25 g) and ingest.	Eliminating phlegm and soften indurated mass, relieving convulsion and spasm, relieve gastric hyperacidity to alleviate stomach ache; Use to treat stomach pain, scrofula, spastic hand and foot.
*R. venosa* (Valenciennes)	same as *R. bezoar*	same as *R. bezoar*
**Genus *Thais***		
*T. alouina* (Röding) ^6^ *T. armiger* (Link) ^7^ *T. bronni* (Dunker) ^8^ *T. bufo* (Lamarck) ^9^ *T. clavigera* (Kuster) ^10^ *T. echinata* (Blainville) ^11^	Decoct the shell (15–50 g) and ingest; used for making pills or medicinal powder; Ustulate the shell, ground into powder and apply externally.	Soften hard lumps, dispel nodes, clear heat, expel toxins; Use to treat pyogenic infection, swelling and ulcer on the body surface and scrofula.
*T. gradate* (Jonas) ^12^	Decoct the crushed shell (15–25 g)	Soften hard lumps, dispel nodes, clear heat, expel toxins, clear expectoration, relieve cough, removing nebula to improve eyesight; Use to treat scrofula, phlegm and cough, scrofula, goitre, *nebula*, swelling and ulcer on the body surface.
*T. hippocastanum* ^13^	Same as *T. alouina*, *etc.*	Same as *T. alouina*, *etc.*
*T. luteostoma* ^14^	Same as *T. gradate*	Same as *T. gradate*
*T. mutabilis* ^15^	Same as *T. alouina*, *etc.*	Same as *T. alouina*, *etc.*
*T. tuberosa* ^16^	Same as *T. alouina*, *etc.*	Same as *T. alouina*, *etc.*

^1^ The information of traditional uses are mainly retrieved from “Zhong Hua Ben Cao” or “The Chinese herbal” [23] and “Zhong Hua Hai Yang Ben Cao” [22]. Based on WoRMS, the accepted name should be ^2^ *Murex aduncospinosus* Sowerby II; ^3^ *Murex pecten pecten* Lightfoot; ^4^ *Vokesimurex rectirostris* (G.B. Sowerby II); ^5^ *Nassa francolina* (Bruguière); ^6^ *Mancinella alouina* (Röding); ^7^ *Mancinella armigera* (Link); ^8^ *Reishia bronni* (Dunker); ^9^ *Purpura bufo* (Lamarck); ^10^ This name is not found in WoRMS. However, it is also named as *Purpura clavigera* (Kuster). Based on WoRMS, the accepted name should be *Reishia clavigera* (Küster); ^11^ *Mancinella echinata* (Blainville); ^12^ Not found in WoRMS. It is also named as *Purpura gradate*, *Purpura trigona* (Reeve) and *Thais trigona* (Reeve). Only *Purpura trigona* (Reeve) was found in WoRMS. The accepted name should be *Indothais gradata* (Jonas); ^13^ *Thais* (*Thalessa*) *virgata* (Dillwyn); ^14^ *Reishia luteostoma* (Holten); ^15^ *Indothais lacera* (Born); ^16^ *Menathais tuberosa* (Röding).

The taxonomy of Muricidae species used in TCM are confusing, as many of the species names used in the two Chinese books “The Chinese herbal” and “Chinese Marine Materia Medica” are synonyms (not accepted) in World Register of Marine Species (WoRMS) (http://www.marinespecies.org/, last access 23/04/2015) (Table 7). Based on the genus names listed in “Chinese Marine Materia Medica”, there are seven genera (*Ceratostoma*, *Chicoreus*, *Murex*, *Nassa*, *Purpura*, *Rapana*, *Thais*) in the Muricidae family used in TCM. Since many synonyms have been used in the Chinese book, there are actually five additional genera (*Indothais*, *Mancinella*, *Menathais*, *Reishia*, *Vokesimurex*) of Muricidae family listed for use in TCM (Table 7). Similar to other traditional medicine, two or more Muricidae species within the same or different genera are often treated as the same TCM medicine, and used interchangeably for the same purpose (Table 7). Only the fresh meat of *T. bufo*, *R. bezoar* and *R. venosa* were claimed to be edible, which may be due to the prevalent toxicity of the choline esters in other Muricidae species, or perhaps just due to their small size.

The most used body part of Muricidae species in TCM is the shell, although there are some records about uses of the fresh meat and operculum of *R. bezoar* and *R. venosa* (Table 7) [11]. The shell is often made into decoctions (extraction by boiling) and ingested, or ustulated (seared or burned) and ground into a powder to apply externally. The medicinal uses of Muricidae species are very diverse, including treating conditions such as insomnia, amnesia, spermatorrhea, uterine bleeding, leukorrhagia, strangury, stomach pain, dyspepsia, stomach and duodenal ulcer, dysentery, scrofula, goitre, pathopyretic ulcer, carbuncle, furuncle, otitis medium, ophthalmalgia, nebula, chronic osteomyelitis, spastic muscles, hepatic coma, phlegm and cough. These claimed therapeutic effects in TCM may be contributed by some of the biologically active chemical compounds in Muricidae species described above. Calcium carbonate from the shell may help alleviate stomach acidity directly, but may also provide a medium for the delivery of other trace Muricidae bioactive compounds, such as bromoisatin and indirubin. However, no studies have specifically investigated the bioactive components in the shell of these snails, or the specific TCM medicines containing Muricidae preparations. Nevertheless, there is some commonality between TCM and the Ancient Mediterranean and Middle Eastern uses of these snails for treatment of stomach pain, ulcers, tumors and eye problems. Of particular interest is the reoccurring use of Muricidae in different cultures to treat menstrual problems, including uterine pain and leukorrhea. These traditional uses may have provided the foundation for the Murex homeopathic remedy.

### 5.3. The Murex Homeopathic Remedy

Despite much controversy over their use [157], globally there remains a widespread, growing market for homeopathic medicines [158]. A homeopathic remedy manufactured from the purple dye secretions of the hypobranchial glands from *Hexaplex* (Murex) *trunculus* and *Bolinus* (Murex, Purpurea) *brandaris* is regularly prescribed by clinical homeopaths and has been part of the homeopathic Materia Medica since the late 1800 s [16,159]. Homeopathic remedies are considered more potent with higher dilution and succession (vigorous shaking). The Murex remedy is typically prescribed at 30C (dilution factor of 10^−60^) or 100C dilution [160]. At such high dilutions it is very unlikely that any of the bioactive compounds found in the hypobranchial glands remain (*i.e*., well above Avogadro’s number). Although homeopathic medicines are generally considered to be safe when administered at such ultra-high dilutions, toxicological aspects must be considered when using lower dilutions or the mother tincture [158]. Traces of 6-bromoisatin have been detected in some batches of the 8X (10^−8^) dilution [104], and both 8X and 4C (10^−4^) Murex remedy can be purchased by any member of the public over the internet.

Murex remedy is prescribed for a range of women’s health complaints from dysmenorrhea (reoccurring severe cramps and pain associated with menstruation) and premenstrual syndrome, to uterine cancer and prolapsed uterus [160,161]. Information about when to prescribe a particular homeopathic remedy is elicited in a process known as proving. These now involve a double blind and placebo controlled methodology, but in the nineteenth century, when information about the therapeutic use of homeopathic Murex was discovered, the proving involved giving a low dilution preparation (e.g., 4C) to a small group of healthy women over 1–2 weeks and meticulously recording changes in their physical, mental and general well-being. The early provings with *Murex* were conducted by Petroz and Hering between 1841 and 1852 on seven women using the 4C dilution, with a further 2 women tested using the 200C dilution by Hering [24,162,163]. These provings are regarded as incomplete and have been further criticized because not all of the subjects were healthy [24]. Nonetheless, the provings elicited some indications for the Murex remedy which have been relied on ever since, including inter-menstrual bleeding, postmenopausal vaginal bleeding and discharge, as well as various abdominal pains and a sensation of heaviness in the uterus, particularly in women who are “highly sexed”, or have “crosswise pain, running from right ovary to left breast” and “profuse perspiration during menses” [162,164]. Lesser [163] points out that most of these symptoms were each only recorded by a single “prover”, although clinical experience of symptoms and conditions that have responded to Murex have been documented and add to the understanding of this homeopathic remedy [164,165].

Overall, there is currently a lack of scientific evidence to substantiate either the symptoms associated with Murex remedy in healthy women, or the efficacy of the treatment in unhealthy women. Despite being sourced from the hypobranchial gland of Muricidae where many biologically active compounds occur, the Murex homeopathic remedy is used at such high serial dilutions that it is very unlikely that any bioactive molecules remain in the therapeutic preparations. People with life-threatening conditions who choose homeopathy may put their health at risk if they reject or delay treatments for which there is proper scientific evidence for safety and effectiveness [158]. Consequently, future studies should consider the potential for developing a biologically active, alternative to the Murex remedy that can be subject to quality control and clinical testing for the treatment of Women’s problems. This may involve trialling nutraceutical formulations containing non-toxic doses of Muricidae hypobranchial gland extracts, containing muscle relaxing, pain killing choline esters, anti-inflammatory and analgesic isatins and the anticancer and steroidogenic modulating indoles.

## 6. Conclusions

Overall there is good evidence that the Muricidae family of marine molluscs produce secondary metabolites with a range of pharmacologically interesting properties. They also have a long history of use in traditional medicines across a number of human cultural groups. However, these traditional medicines are relatively rarely used and have never been tested for efficacy in clinical trials. Furthermore, the specific decoctions prepared from Muricidae for medicinal purposes have not been chemically analysed. Nevertheless, there are some interesting commonalities in the traditional therapeutic uses and the bioactive properties of Muricidae that warrant further investigation. In particular, preparations used for wound healing, inflammation, pain and menstrual problems should be investigated for biologically active concentrations of indole derivatives and choline esters.

The species specificity and relatively rare use of the whole Muricid body in traditional medicines could be in part explained by the relative composition of choline esters, such as the toxic tigloylcholine relative to murexine. The efficacy of different species may also be influenced by the specific indoxyl sulfate precursors present and the proportion that are brominated, as well as the oxidative reactions and stabilisation of compounds in the different therapeutic preparations. Given the development of modern molecular methods for Muricidae taxonomy and analytical chemistry procedures for separating, identifying and quantifying the bioactive compounds, it should be possible to optimise the selection and extraction of Muricidae species for the development of an improved natural medicine. As for all complementary and alternative medicines, any new Muricidae nutraceutical would need to be subjected to rigorous scientific efficacy and safety testing and monitored for quality control.

## References

[1] Newman D.J., Cragg G.M. (2004). Marine natural products and related compounds in clinical and advanced preclinical trials. J. Nat. Prod..

[2] Simmons T.L., Andrianasolo E., McPhail K., Flatt P., Gerwick W.H. (2005). Marine natural products as anticancer drugs. Mol. Can. Ther..

[3] Benkendorff K. (2010). Molluscan biological and chemical diversity: Secondary metabolites and medicinal resources produced by marine molluscs. Biol. Rev..

[4] Mayer A.M., Glaser K.B., Cuevas C., Jacobs R.S., Kem W., Little R.D., McIntosh J.M., Newman D.J., Potts B.C., Shuster D.E. (2010). The odyssey of marine pharmaceuticals: A current pipeline perspective. Trends Pharmacol. Sci..

[5] Blunt J.W., Copp B.R., Keyzers R.A., Munro M.H.G., Prinsep M.R. (2012). Marine natural products. Nat. Prod. Rep..

[6] Benkendorff K., Di Cosmo A., Winlow W. (2014). Chemical diversity in molluscan communities: From natural products to chemical ecology. Neuroecology and Neuroethology in Molluscs: The interface Between Behaviour and Environment.

[7] Madden T., Tran H.T., Beck D., Huie R., Newman R.A., Pusztai L., Wright J.J., Abbruzzese J.L. (2000). Novel marine-derived anticancer agents: A phase I clinical, pharmacological, and pharmacodynamic study of dolastatin 10 (nsc 376128) in patients with advanced solid tumors. Clin. Cancer. Res..

[8] Chin Y., Balunas M.J., Chai H.B., Kinghorn D. (2006). Drug discovery from natural sources. AAPS J..

[9] Prabhakar A.K., Roy S.P. (2009). Ethno-medicinal uses of some shell fishes by people of Kosi River basin of North-Bihar, India. Stud. Ethno-Med..

[10] Herbert D.G., Hamer M.L., Mander M., Mkhize N., Prins F. (2003). Invertebrate animals as a component of the traditional medicine trade in Kwazulu- Natal, South Africa. Afr. Invert..

[11] Yang X.R. (2003). Encyclopedic Reference of Traditional Chinese Medicine.

[12] Gopal R., Vijayakumaran M., Venkatesam R., Kathiroli S. (2008). Marine organisms in Indian medicine and their future prospects. Nat. Prod. Rad..

[13] Meyerhof M., Sobhy G.P. (1932). The Abridged Version of “The Book of Simple Drugs”, of Ahmad Ibn Muhammad Al-Ghafiqi by Gregorius Abul-Farag.

[14] Lev E., Zohar A. (2008). Practical Materia Medica of the Medieval Eastern Mediterranean According to the Cairo Genizah.

[15] Levey M. (1961). Ibn Mäsawaih and his treatise on simple aromatic substances: Studies in the history of Arabic pharmacology I. J. His. Med. All. Sci..

[16] HPUS (1878). Homœopathic Pharmacopoeia of the United States.

[17] Andlauer W., Furst P. (2002). Nutraceuticals: A piece of history, present status and outlook. Food Res. Int..

[18] Barrow C., Shahidi F. (2007). Marine Nutraceuticals and Functional Foods.

[19] Piscitelli S.C., Burstein A.H., Chaitt D., Alfaro R.M., Falloon J. (2000). Indinavir concentrations and St John’s wort. Lancet.

[20] Straus S.E. (2000). Complementary and alternative medicine: Challenges and opportunities for pharmacology and therapeutic research. Pharmacologist.

[21] Voultsiadou E. (2010). Therapeutic properties and uses of marine invertebrates in the ancient greek world and early byzantium. J. Ethnopharm..

[22] Guan H.S., Wang S.G. (2009). Hai yang wu ji zhui dong wu yao. Zhong Hua Hai Ben Cao (Chinese Marine Materia Medica).

[23] China State Administration Traditional Chinese Materia Medica Editorial Board (1999). Zhong Hua Ben Cao (The Chinese Materia Medica).

[24] Dunham C. (1864). Murex purpurea. Am. Homeo. Rev..

[25] Benkendorff K. (2013). Natural product research in the Australian marine invertebrate *Dicathais orbita*. Mar. Drugs.

[26] Dolashka P., Voelter W. (2013). Antiviral activity of hemocyanins. Invertebr. Surviv. J..

[27] Barco A., Claremont M., Reid D.G., Houart R., Bouchet P., Williams S.T., Cruaud C., Couloux A., Oliverio M. (2010). A molecular phylogenetic framework for the Muricidae, a diverse family of carnivorous gastropods. Mol. Phylogenet. Evol..

[28] Jennings S., Kaiser M.J., Reynolds J.D. (2001). Marine Fisheries Ecology.

[29] Leiva G.E., Castilla J.C. (2001). A review of the world marine gastropod fishery: Evolution of catches, management and the Chilean experience. Rev. Fish Biol. Fish..

[30] Vasconcelos P., Carvalho S., Castro M., Gaspar M. (2008). The artisanal fishery for muricid gastropods (banded murex and purple dye murex) in the Ria Formosa Lagoon (Algrave Coast, Southern Portugal). Sci. Mar..

[31] Woodcock S.H., Benkendorff K. (2008). The impact of diet in the growth and proximate composition of juvenile whelks, *Dicathais orbita* (Gastropoda: Mollusca). Aquaculture.

[32] Mchugh J. (2013). Blattes de byzance in India: Mollusk opercula and the history of perfumery. J. Roy. Asia. Soc..

[33] Nawata H., Fukui K., Kurimo E. (1997). An exported item from Badi on the western Red Sea coast in the eighth century: Historical and ethnographical studies on operculum as incense and perfume. Ethiopia in Broader Perspective: ICES: International Conference of Ethiopian Studies.

[34] Cooksey C.J. (2001). Tyrian purple: 6,6′-dibromoindigo and related compounds. Molecules.

[35] Naegel L.C.A., Cooksey C.J. (2002). Tyrian purple from marine muricids, especially from *Plicopurpura pansa* (Gould, 1853). J. Shellfish Res..

[36] Cooksey C. (2013). Tyrian purple: The first four thousand years. Sci. Prog..

[37] López-Chávez F.J., Ríos-Chávez P., Oymam K. (2009). Brominated precursors of Tyrian purple (C.I. Natural violet 1) from *Plicopupura pansa*, *Plicopurpura columellaris* and *Plicopurpura patula*. Dye Pigment..

[38] Baker J.T. (1974). Tyrian purple: An ancient dye, a modern problem. Endeavour.

[39] Jensen L.B. (1963). Royal purple of Tyre. J. Near East. Stud..

[40] Allan J.K. (1934). Tyrian purple: An ancient industry. Aust Mus. Mag..

[41] Hutchinson R.W. (1962). Prehistoric Crete.

[42] McGovern P.E., Michel R.H. (1990). Royal purple dye: The chemical reconstruction of the ancient Mediterranean industry. Am. Chem. Soc..

[43] Naegel L.C.A., Alvarez J.I.M. (2005). Biological and chemical properties of the secretion from the hypobranchial gland of the purple snail *Plicopurpura pansa* (Gould 1853). J. Shellfish Res..

[44] Sterman B., Taubes-Sterman J. (2012). The Rarest Blue.

[45] Cooksey C.J., Meijer L., Guyard N., Skaltsounis L., Eisenbrand G. (2006). Marine indirubins. Indirubin, the Red Shade of Indigo.

[46] Nawata H. (2001). Coastal resource use by camel pastoralists: A case study of gathering and fishing activities among the Beja in Eastern Sudan. Nilo-Ethiop. Stud..

[47] Pulak C., Aruz J., Bensel K., Evans J. (2008). The Uluburun shipwreck and late bronze age trade. Beyond Babylon: Art, Trade, and Diplomacy in the Second Millennium B.C..

[48] Friedlander P., Bruckner S., Deutsch G. (1906). Bromo-and methoxy derivatives of indigo. Justus Liebigs Annalen der Chemie.

[49] Hamburger M. (2002). *Isatis tinctoria*—From the rediscovery of an ancient medicinal plant towards a novel anti-inflammatory phytopharmaceutical. Phytochem. Rev..

[50] Lim H., Chung E., Kin J.-C., Choi G., Jang K., Chung Y., Cho Y., Lee S.-W. (2005). Characterization of a forest soil metagenome clone that confers indirubin and indigo production on *Escherichia coli*. Appl. Envi. Micro..

[51] Westley C.B., Vine K.L., Benkendorff K., Meijer L., Guyard N., Skaltsounis L., Eisenbrand G. (2006). A proposed functional role for indole derivatives in reproduction and defence of the Muricidae (Neogastropoda: Mollusca). Indirubin, the Red Shade of Indigo.

[52] Fouquet H., Bielig H.J. (1971). Biological precursors and genesis of Tyrian purple. Angew. Chem..

[53] Baker J.T., Duke C.C. (1973). Isolation from the hypobranchial glands of marine molluscs of 6-bromo-2,2-dimethylthioindolin-3-one and 6-bromo-2-methylthioindoleninone as alternative precursors to Tyrian purple. Tetrahedron Lett..

[54] Baker J., Duke C. (1976). Isolation of choline and choline ester salts of tyrindoxyl sulphate from the marine molluscs *Dicathais orbita* and *Mancinella keineri*. Tetrahedron Lett..

[55] Baker J., Duke C. (1973). Chemistry of the indoleninones. Ii. Isolation from the hypobranchial glands of marine molluscs of 6-bromo-2,2-dimethylthioindolin-3-one and 6-bromo-2-methylthioindoleninone as alternative precursors to Tyrian purple. Aust. J. Chem..

[56] Baker J.T., Duke C.C. (1974). Precursors of tyrian purple. Food-Drugs Sea Pro..

[57] Baker J., Sutherland M. (1968). Pigments of marine animals VIII. Precursors of 6,6′-dibromoindigotin (tyrian purple) from the mollusc *Dicathais orbita* (Gmelin). Tetrahedron Lett..

[58] Benkendorff K., Bremner J.B., Davis A.R. (2000). Tyrian purple precursors in the egg masses of the Australian muricid, *Dicathais orbita*: A possible defensive role. J. Chem. Ecol..

[59] Palma H., Paredes J., Cristi E. (1999). 6,6′-Dibromoindigotin en capsulas de embriones di *Concholepas concholepas* (Bruguiere, 1789). Medio Ambient..

[60] Benkendorff K., Westley C.B., Gallardo C.S. (2004). Observations on the production of purple pigments in the egg capsules, hypobranchial and reproductive glands from seven species of Muricidae (Gastropoda: Mollusca). Invert. Reprod. Dev..

[61] Westley C., Benkendorff K. (2008). Sex-specific tyrian purple genesis: Precursor and pigment distribution in the reproductive system of the marine mollusc, *Dicathais orbita*. J. Chem. Ecol..

[62] Benkendorff K., Bremner J., Davis A. (2001). Indole derivatives from the egg masses of muricid molluscs. Molecules.

[63] Benkendorff K., Pillai R., Bremner J.B. (2004). 2,4,5-tribromo-1 h-imidazole in the egg masses of three muricid molluscs. Nat. Prod. Res..

[64] Ochi N., Kataoka K., Ariki S., Iwatsuki C., Kodama M., Fukuyama Y. (1998). Antioxidative bromoindole derivatives from the mid-intestinal gland of the muricid gastropod *Drupella fragum*. J. Nat. Prod..

[65] Fukuyama F., Iwatsuki C., Kodama M., Ochi M., Kataoka K., Shibata K. (1998). Antimicrobial indolequinones from the mid-intestinal gland of the muricid gastropod *Drupella fragum*. Tetrahedron.

[66] Gul W., Hamann M.T. (2005). Indole alkaloid marine natural products: An established source of cancer drug leads with considerable promise for hte control of parasitic, neurological and other diseases. Life Sci..

[67] Vine K.L., Matesic L., Locke J.M., Ranson M., Skropeta D. (2009). Cytotoxic and anticancer activities of isatin and its derivatives: A comprehensive review from 2000–2008. Anticancer Agents Med. Chem..

[68] Esmaeelian B., Benkendorff K., Johnston M., Abbott C. (2013). Purified brominated indole derivatives from *Dicathais orbita* induce apoptosis and cell cycle arrest in colorectal cancer cell lines. Mar. Drugs.

[69] Edwards V., Benkendorff K., Young F. (2012). Marine compounds selectively induce apoptosis in female reproductive cancer cells but not in primary-derived human reproductive granulosa cells. Mar. Drugs.

[70] Rudd D., Benkendorff K. (2014). Supercritical CO_2_ extraction of bioactive Tyrian purple precursors from the hypobranchial gland of a marine gastropod. J. Supercrit. Fluids.

[71] Ronci M., Rudd D., Guinan T., Benkendorff K., Voelcker N. (2012). Mass spectrometry imaging on porous silicon: Investigating the distribution of bioactives in marine mollusc tissues. Anal. Chem..

[72] Rudd D., Benkendorff K., Voelcker N.H. (2015). Solvent separating secondary metabolites directly from biosynthetic tissue for surface-assisted laser desorption ionisation mass spectrometry. Mar. Drugs.

[73] Roseghini M., Erspamer V., Ramorino L., Gutierrez J.E. (1970). Choline esters, their precursors and metabolites in the hypobranchial gland of prosobranchiate molluscs, *Concholepas concholepas* and *Thais chocolata*. Eur. J. Biochem. FEBS.

[74] Roseghini M., Severini C., Erspamer G.F., Erspamer V. (1996). Choline esters and biogenic amines in the hypobranchial gland of 55 molluscan species of the neogastropod Muricoidea superfamily. Toxicon.

[75] Erspamer V., Benati O. (1953). Identification of murexine as beta-(imidazolyl-(4))-acrylcholine. Science.

[76] Bender J.A., Deriemer K., Roberts T.E., Rushton R., Boothe P., Mosher H.S., Fuhrman F.A. (1974). Choline esters in marine gastropods *Nucella emarginata* and *Acanthina spirata*—New choline ester, tentatively identified as *N*-methylmurexine. General Pharmacol..

[77] Shiomi K., Ishii M., Shimakura K., Nagashima Y., Chino M. (1998). Tigloylcholine: A new choline ester toxin from the hypobranchial gland of two species of muricid gastropods (*Thais clavigera* and *Thais bronni*). Toxicon.

[78] Duke C.C., Eichholzer J.V., Macleod J.K. (1978). *N*-methylmurexine—Naturally occuring marine compound. Tetrahedron Lett..

[79] Ramasamy M.S., Murugan A. (2005). Potential antimicrobial activity of marine molluscs from Tuticorin, southeast coast of India against 40 biofilm bacteria. J. Shellfish Res..

[80] D’Armas H., Yáñez D., Reyes D., Salazar G. (2010). Composición de ácidos grasos de los caracoles marinos *Phyllonotus pomum* y *Chicoreus brevifrons* (gastropoda: Muricidae). Rev. Biol. Trop..

[81] Zarai Z., Frikha F., Balti R., Miled N., Gargouri Y., Mejdoub H. (2011). Nutrient composition of the marine snail (*Hexaplex trunculus*) from the Tunisian Mediterranean coasts. J. Sci. Food Agric..

[82] Benkendorff K., Davis A.R., Rogers C.N., Bremner J.B. (2005). Free fatty acids and sterols in the benthic spawn of aquatic molluscs, and their associated antimicrobial properties. J. Exp. Mar. Biol. Ecol..

[83] Benkendorff K., Davis A.R., Bremner J.B. (2001). Chemical defense in the egg masses of benthic invertebrates: An assessment of antibacterial activity in 39 mollusks and 4 polychaetes. J. Invert. Path.

[84] Benkendorff K. (1999). Bioactive Molluscan Resources and Their Conservation: Biological and Chemical Studies on the Egg Masses of Marine Molluscs. Ph.D. Thesis.

[85] Dolashka P., Moshtanska V., Borisova V., Dolashki A., Stevanovic S., Dimanov T., Voelter W. (2011). Antimicrobial proline-rich peptides from the hemolymph of marine snail *Rapana venosa*. Peptides.

[86] Dolashka P., Nesterova N., Zagorodnya S., Dolashki A., Baranova G., Golovan A.W.V. (2014). Antiviral activity of hemocyanins *Rapana venosa* and its isoforms against Epstein-Barr virus. Glob. J. Pharm..

[87] Velkova L., Todorov D., Dimitrova I., Shishkov S., Van Beeumen J., Dolashka-Angelova P. (2009). *Rapana venosa* hemocyanin with antiviral activity. Biotech. Biotech. Equip..

[88] WoRMS Editorial Board. World Register of Marine Species.

[89] Ivanov M., Todorovska E., Radkova M., Georgiev O., Dolashki A., Dolashka P. (2015). Molecular cloning, characterization and phylogenetic analysis of an actin gene from the marine mollusk *Rapana venosa*. Int. J. Curr. Microbiol. App. Sci..

[90] Badiu D.L., Balu A.M., Barbes L., Luque R., Nita R., Radu M., Tanase E., Rosoiu N. (2008). Physico-chemical characterisation of lipids from *Mytilus galloprovincialis* (L.) and *Rapana venosa* and their healing properties on skin burns. Lipids.

[91] Badiu D.L., Luque R., Dumitrescu E., Craciun A., Dinca D. (2010). Amino acids from *Mytilus galloprovincialis* (L.) and *Rapana venosa* molluscs accelerate skin wounds healing via enhancement of dermal and epidermal neoformation. Protein J..

[92] Becker M., Fuentes A., Del Campo M., Manubens A., Nova E., Oliva H., Faunes F., Valenzuela M., Campos-Vallette M., Aliaga A. (2009). Immunodominant role of CCHA subunit of *Concholepas* hemocyanin is associated with unique biochemical properties. Int. Immunopharmacol..

[93] Tchorbanov A., Idakieva K., Mihaylova N., Doumanova L. (2008). Modulation of the immune response using *Rapana thomasiana* hemocyanin. Int. Immunopharmacol..

[94] Cantillana P., Inestrosa N.C. (1991). Presence of a heparin-binding growth factor in *Concholepas concholepas* Bruguiere (Mollusca: Gastropoda; Muricidae). J. Exp. Mar. Bio. Ecol..

[95] Man Y., Wang Y.-X., ZHu S.-Y., Zhao D., Hu F., Lu J.-Y. (2012). Indirubin inhibits ATP-induced phagocytosis attenuation, ROS production and cell death of macrophages. Acta Pharm. Sin..

[96] Kim J.K., Park G.M. (2012). Indirubin-3-monoxime exhibits anti-inflammatory properties by down-regulating NF-kappab and JNK signaling pathways in lipopolysaccharide-treated RAW264.7 cells. Inflamm. Res..

[97] Jung H.-J., Nam K.N., Son M.-S., Kang H., Hong J.-W., Kim J.W., Lee E.H. (2011). Indirubin-3′-oxime inhibits inflammatory activation of rat brain microglia. Neurosci. Lett..

[98] Matheus M.E., Flavio de Almeida V., Garden S., Pinto J., Fernamdes A.C., Dias P. (2007). Isatins inhibit cyclooxygenase-2 and inducible nitric oxide synthase in a mouse macrophage cell line. Eur. J. Pharmacol..

[99] Kitaura Y., Ito F., Stevens R.W., Asai N. (1990). Antiallergy and Antiinflamatory Agents. U.S. Patent.

[100] Stevens R.W., Morita H., Nakane M. (1994). Indole Derivatives as Antiallergy and Antiinflammatory Agents. U.S. Patent.

[101] Olofsson K., Suna E., Pelcman B., Ozola V., Kakvins I., Schaal W. (2006). Indoles Useful in the Treatment of Inflammation. U.S. Patent.

[102] Farina C., Gagliardi S., Novella P.A. (2004). Indole Derivatives and Their Use for the Treatment of Osteoporosis amongst Other Applications. U.S. Patent.

[103] Yazback R., Lindsay R., Abbott C.A., Benkendorff K., Howarth G.S. (2015). Combined effects of muricid extract and 5-fluorouracil on intestinal toxicity in rats. Evid.-Based Complement. Altern. Med..

[104] Benkendorff K., McIver C.M., Abbott C.A. (2011). Bioactivity of the murex homeopathic remedy and of extracts from an Australian muricid mollusc against human cancer cells. Evid.-Based Complement. Altern. Med..

[105] Vine K., Locke J., Ranson M., Benkendorff K., Pyne S., Bremner J. (2007). *In vitro* cytotoxicity evaluation of some substituted isatin derivatives. Bioorg. Med. Chem..

[106] Edwards V., Young F., Benkendorff K. (2014). An *in vitro* high throughput assay for screening reproductive and toxic effects of anticancer compounds. Biotech. Appl. Biochem..

[107] Meijer L., Shearer J., Bettayeb K., Ferandin Y., Meijer L., Guyard N., Skaltsounis A.-L., Eisenbrand G. (2006). Diversity of intracellular mechanisms underlying the anti-tumor properties of indirubins. Indirubin, the Red Shade of Indigo.

[108] Magiatis P., Skaltsounis A.L., Meijer L., Guyard N., Skaltsounis A.-L., Eisenbrand G. (2006). From *Hexaplex trunculus* to new kinase inhibitory indirubins. Indirubin, the Red shade of Indigo.

[109] Zhang L., Fan X., Han L. (2005). Antitumor and immune regulation activities of the extracts of some chinese marine invertebrates. Chin. J. Oceanol. Limnol..

[110] Genova-Kalou P., Idakieva K., Dundarova D., Argirova R., Alexandrova R., Yotovska K., Mohmmed A. (2008). Cytotoxic and apoptogenic properties of the hemocyanin derived from the marine mollusk *Rapana thomasiana in vitro*. Planta Med..

[111] Antonova O., Yossifova L., Staneva R., Stevanovic S., Dolashka P., Toncheva D. (2015). Changes in the gene expression profile of the bladder cancer cell lines after treatment with *Helix lucorum* and *Rapana venosa* hemocyanin. J. Balk. Union Oncol..

[112] Esmaeelian B., Abbott C., Le Leu R., Benkendorff K. (2014). 6-bromoisatin found in muricid mollusc extracts inhibits colon cancer cell proliferation and induces apoptosis, preventing early stage tumor formation in a colorectal cancer rodent model. Mar. Drugs.

[113] Meijer L., Skaltsounis A.L., Magiatis P., Polychronopoulos P., Knockaert M., Leost M., Ryan X.P., Vonica C.A., Brivanlou A., Dajani R. (2003). GSK-3-selective inhibitors derived from Tyrian purple indirubins. Chem. Biol..

[114] Hoessel R., Leclerc S., Endicot J.A., Nobel M.E.M., Lawrie A., Tunnah P., Leost M., Damiens E., Marie D., Marko D. (1999). Indirubin, the active constituent of a chinese antileukaemia medicine, inhibits cyclin-dependent kinases. Nat. Cell Biol..

[115] Leclerc S., Garnier M., Hoessel R., Marko D., Bibb J.A., Snyder G.L., Greengard P., Jacek Biernati J., Wui Y., Mandelkowi E. (2001). Indirubins inhibit glycogen synthase kinase-3b and CDK5/p25, two protein kinases involved in abnormal tau phosphorylation in alzheimer’s disease. J. Biol. Chem..

[116] Vougogiannopoulou K., Skaltsounis A.L. (2012). From Tyrian purple to kinase modulators: Naturally halogenated indirubins and synthetic analogues. Planta Med..

[117] Saito H., Tabata K., Hanada S., Kanda Y., Suzuki T., Miyairi S. (2011). Synthesis of methoxy- and bromo-substituted indirubins and their activities on apoptosis induction in human neuroblastoma cells. Bioorg. Med. Chem. Lett..

[118] Nicolaou K.A., Liapis V., Evdokiou A., Constantinou C., Magiatis P., Skaltsounis A.L., Koumas L., Costeas P.A., Constantinou A.I. (2012). Induction of discrete apoptotic pathways by bromo-substituted indirubin derivatives in invasive breast cancer cells. Biochem. Biophys. Res. Commun..

[119] Liu L., Nam S., Tian Y., Yang F., Wu J., Wang Y., Scuto A., Polychronopoulos P., Magiatis P., Skaltsounis L. (2011). 6-bromoindirubin-3′-oxime inhibits JAK/STAT3 signaling and induces apoptosis of human melanoma cells. Cancer Res..

[120] Choi S.J., Moon M.J., Lee S.D., Choi S.U., Han S.Y., Kim Y.C. (2010). Indirubin derivatives as potent FLT3 inhibitors with anti-proliferative activity of acute myeloid leukemic cells. Bioorg. Med. Chem. Lett..

[121] Edwards V., Young F., Benkendorff K. (2013). Effects of Murcidae extracts on estrogen-sensitive breast cancer and the steroidogenic pathway.

[122] Grubb G.S., Fensome A., Miller L.L., Ullrich J.W., Bender R.H.W., Zhang P., Wrobel J.E., Edwards J.P., Jones T.K., Tegley C.M. (2003). Cyclic Regimens Utilizing Indoline Derivatives. U.S. Patent.

[123] Westley C.B., McIver C.M., Abbott C.A., Le Leu R.K., Benkendorff K. (2010). Enhanced acute apoptotic response to azoxymethane-induced DNA damage in the rodent colonic epithelium by tyrian purple precursors a potential colorectal cancer chemopreventative. Cancer Biol. Ther..

[124] Esmaeelian B. (2014). *In-Vitro* and *in Vivo* Testing of Purified Muricid Mollusc Extract on Colorectal Cancer. Ph.D. Thesis.

[125] Fiebig H.H., Schuler J., Meijer L., Guyard N., Skaltsounis A.L., Eisenbrand G. (2006). *In vivo* anti-tumour activity of indirubins. Indirubin, the Red Shade of Indigo.

[126] Wang R. (2009). Effects of Marine Mollusc Extracts on Human Immune Function. Master’s Thesis.

[127] Cane A., Tournaire M.-C., Barritault D., Crumeyrolle-Arias M. (2000). The endogenous oxindoles 5-hydroxyoxindole and isatin are antiproliferative and proapoptotic. Biochem. Biophys. Res.Commun..

[128] Igosheva N., Lorz C., O’Conner E., Glover V., Mehmet H. (2005). Isatin, an endogenous monoamine oxidase inhibitor, triggers a dose-and time-dependent switch from apoptosis to necrosis in human neuroblastoma cells. Neurochem. Int..

[129] Gaboriaud-Kolar N., Vougogiannopoulou K., Skaltsounis A.-L. (2014). Indirubin derivatives: A patent review (2010-present). Expert Opin. Ther. Pat..

[130] Nam S., Buettner R., Turkson J., Kim D., Cheng J.Q., Muehlbeyer S., Hippe F., Vatter S., Merz K.H., Eisenbrand G. (2005). Indirubin derivatives inhibit STAT3 signaling and induce apoptosis in human cancer cells. PNAS.

[131] Kim S.A., Kim Y.C., Kim S.W., Lee S.H., Min J.J., Ahn S.G., Yoon J.H. (2007). Antitumor activity of novel indirubin derivatives in rat tumor model. Clin. Cancer Res..

[132] Karabelas K., Lepisto M., Sjo P. (2002). Indoles as Protein Kinase Inhibitors. U.S. Patent.

[133] Kim Y.C., Kim S.W., Kim T.S., Lee S.K., Kim J.D., Yoon J.H. (2009). Indirubin Derivatives Having Anticancer Property against Human Cancer Cell Lines. U.S. Patent.

[134] Wang L., Liu X.P., Chen R. (2005). Derivatives of Isoindigo, Indigo and Indirubin and Methods of Treating Cancer. U.S. Patent.

[135] Wang L., Liu X.P., Chen R. (2003). Derivative of Isoindigo and Indirubin for the Treatment of Cancer. U.S. Patent.

[136] Carson D.A., Leoni L.M., Cotton H.B. (2006). Indoles Treatment of Cancer. U.S. Patent.

[137] Leung P.C.K., Adashi E.Y. (2004). The Ovary.

[138] Edwards V. (2012). The Effects of Bioactive Compounds from the Marine Mollusc *Dicathais Orbita* on Human Reproductive Cells and Human Reproductive Cancer Cells. Ph.D. Thesis.

[139] Ohlsson A., Cedergreen N., Oskarsson A., Ullera E. (2010). Mixture effects of imidazole fungicides on cortisol and aldosterone secretion in human adrenocortical H295r cells. Toxicology.

[140] Erspamer V., Glasser A. (1957). The pharmacological actions of murexine (urocanylcholine). Br. J. Pharmacol. Chemother..

[141] Keyl M.J., Whittaker V.P. (1958). Some pharmacological properties of murexine (urocanoylcholine). Br. J. Pharmacol. Chemother..

[142] Bhattacharya S.K., Mitra S.K., Acharya S.B. (1991). Anxiogenic activity of isatin, a putative biological factor, in rodents. J. Psychopharmacol.

[143] Abel E.L. (1995). Behavioral effects of isatin on open field activity and immobility in the forced swim test in rats. Physiol. Behav..

[144] Bhattacharya S.K., Chakrabarti A. (1998). Dose-related proconvulsant and anticonvulsant activity of isatin, a putative biological factor, in rats. Indian. J. Exp. Biol..

[145] Ahring P.K., Christophersen P., Jensen B.S., Jorgensen T.D., Strobaek D., Teuber L., Olesen S.P. (2000). Use of Isatin Derivatives as Ion Channel Activating Agents. International Patent.

[146] Watjen F., Drejer J., Jensen L.H. (1995). Isatin Derivatives, Their Preparation and Use. European Patent.

[147] Debat J. (1972). Promotion of Analgesic and Sedative Action with 5-Bromoisatin. International Patent.

[148] Hey P. (1952). On relationships between structure and nicotine-like stimulant activity in choline esters and ethers. Br. J. Pharmacol. Chemother..

[149] Medvedev A., Buneeva O., Glover V. (2007). Biological targets for isatin and its analogues: Implications for therapy. Biologics.

[150] Crumeyrolle-Arias M., Medvedev A., Cardona A., Barritault D., Glover V. (2003). *In situ* imaging of specific binding of [H]isatin in rat brain. J. Neurochem..

[151] Medvedev A.E., Sandler M., Glover V. (1998). Interaction of isatin with type-a natriuretic peptide receptor: Possible mechanism. Life Sci..

[152] Westley C.B., Benkendorff K., McIver C.M., Le Leu R.K., Abbott C.A. (2013). Gastrointestinal and hepatotoxicity assessment of an anticancer extract from muricid molluscs. Evid. Based Complement. Altern. Med..

[153] Chahal C.S. (2014). A Study of the Anticancer Properties of the Crude Extract and Synthetic 6-Bromoisatin from *Dicathais orbita*: An *In Vivo* Colorectal Cancer Mice Model. Master’s Thesis.

[154] Nataraj K.S., Rao J.V., Jayaveera K.N. (2010). Diuretic Activity of Some Novel Isatin Derivatives. J. Pharm. Res..

[155] Lev E. (2007). Drugs held and sold by pharmacists of the Jewish community of medieval (11–14th Centuries) Cairo according to lists of *Materia Medica* found at the Taylor–Schechter Genizah collection, Cambridge. J. Ethnopharmacol..

[156] Rätsch C., Müller-Ebeling C. (2013). The Encyclopedia of Aphrodisiacs: Psychoactive Substances for Use in Sexual Practices.

[157] National Health and Medical Research Council (2015). NHMRC Information Paper: Evidence on the Effectiveness of Homeopathy for Treating Health Conditions.

[158] World Health Organisation (2009). Saftey Issues in the Preparation of Homeopathic Medicines.

[159] HPUS Monograph 6116 Murex Purpurea in Homoepathic Pharmacopoeia of the United States. www.hpus.com/online_database/view_monograph_action.php?id=6116.

[160] Zacharia G. (2012). Personal communication.

[161] Lee A. (2011). Homeopathic Mind Maps Remedies of the Animal Kingdom.

[162] Hering C. (1890). Guiding Symptoms of our Materia Medica.

[163] Lesser O. (1990). The molluscs: Murex and Sepia. Br. Homeopath. J..

[164] Vermeulen F. (2002). Prisma.

[165] Murphy R. (2005). Homeopathic Clinical Repertory.

